# Early-Neonatal, Late-Neonatal, Postneonatal, and Child Mortality Rates Across India, 1993-2021

**DOI:** 10.1001/jamanetworkopen.2024.10046

**Published:** 2024-05-10

**Authors:** S. V. Subramanian, Akhil Kumar, Thomas W. Pullum, Mayanka Ambade, Sunil Rajpal, Rockli Kim

**Affiliations:** 1 Harvard Center for Population and Development Studies, Boston, Massachusetts; 2 Department of Social and Behavioral Sciences, Harvard T. H. Chan School of Public Health, Boston, Massachusetts; 3 Faculty of Arts and Sciences, University of Toronto, Toronto, Ontario, Canada; 4 The Demographic and Health Surveys Program, ICF; 5 Department of Sociology, University of Texas, Austin; 6 Indian Institute of Technology, Mandi, Himachal Pradesh, India; 7 Department of Economics, FLAME University, Pune, India; 8 Division of Health Policy and Management, College of Health Science, Korea University, Seoul, South Korea; 9 Interdisciplinary Program in Precision Public Health, Department of Public Health Sciences, Graduate School of Korea University, Seoul, South Korea

## Abstract

**Importance:**

The global success of the child survival agenda depends on how rapidly mortality at early ages after birth declines in India, and changes need to be monitored to evaluate the status.

**Objective:**

To understand the disaggregated patterns of decrease in early-life mortality across states and union territories (UTs) of India.

**Design, Setting, and Participants:**

Repeated cross-sectional data from the 5 rounds of the National Family Health Survey conducted in 1992-1993, 1998-1999, 2005-2006, 2015-2016, and 2019-2021 were used in a representative population-based study. The study was based on data of children born in the past 5 years with complete information on date of birth and age at death. The analysis was conducted in February 2024.

**Exposure:**

Time and geographic units.

**Main Outcomes and Measures:**

Mortality rates were computed for 4 early-life periods: early-neonatal (first 7 days), late-neonatal (8-28 days), postneonatal (29 days to 11 months), and child (12-59 months). For early and late neonatal periods, the rates are expressed as *deaths per 1000 live births*, for postneonatal, as *deaths per 1000 children aged at least 29 days* and for child, *deaths per 1000 children aged at least 1 year*. These are collectively mentioned as *deaths per 1000* for all mortalities. The relative burden of each of the age-specific mortalities to total mortality in children younger than 5 years was also computed.

**Results:**

The final weighted analytical sample included 6879 (1993), 5482 (1999), 4293 (2006), 12612 (2016), and 9788 (2021) children who died before their fifth birthday in the past 5 years of each survey. Mortality rates were lowest for the late-neonatal and child periods; early-neonatal was the highest in 2021. Child mortality experienced the most substantial decrease between 1993 and 2021, from 33.5 to 6.9 deaths per 1000, accompanied by a substantial reduction in interstate inequalities. While early-neonatal (from 33.5 to 20.3 deaths per 1000), late-neonatal (from 14.1 to 4.1 deaths per 1000), and postneonatal (from 31.0 to 10.8 deaths per 1000) mortality also decreased, interstate inequalities remained notable. The mortality burden shifted over time and is now concentrated during the early-neonatal (48.3% of total deaths in children younger than 5 years) and postneonatal (25.6%) periods. A stagnation or worsening for certain states and UTs was observed from 2016 to 2021 for early-neonatal, late-neonatal, and postneonatal mortality. If this pattern continues, these states and UTs will not meet the United Nations Sustainable Development Goal targets related to child survival.

**Conclusions and Relevance:**

In this repeated cross-sectional study of 5 time periods, the decrease in mortality during early-neonatal and postneonatal phases of mortality was relatively slower, with notable variations across states and UTs. The findings suggest that policies pertaining to early-neonatal and postneonatal mortalities need to be prioritized and targeting of policies and interventions needs to be context-specific.

## Key Points

**Question:**

How have the early-neonatal, late-neonatal, postneonatal, and child mortality rates in the 36 states and union territories of India changed over the past 30 years?

**Findings:**

In this repeated cross-sectional study of 39054 children who died before their fifth birthday in the past 5 years of each survey from each of the rounds of the National Family Health Survey, the lowest mortality rates were observed for the late-neonatal and child periods; the early-neonatal period was the highest, followed by the postneonatal period. Assessing change in absolute terms, child mortality decreased the most; the burden of mortality at early ages is increasingly concentrated in the early-neonatal and postneonatal phase.

**Meaning:**

The findings of this study suggest that interventions and resources need to be prioritized according to the disaggregated mortality risk in a given area.

## Teaser

This cross-sectional study evaluates changes in neonatal and child mortality across states and union territories in India from 1993 to 2021.

## Introduction

The Sustainable Development Goals (SDGs) of the United Nations include reducing mortality in the first 5 years to 25 deaths per 1000 live births and the first 28 days to 12 deaths per 1000 live births by 2030.^1,2^ Nearly 5 million children throughout the world died before their fifth birthday in 2021, with 27% living in South Asia.^3,4^ India, with a rate of 42 children younger than 5 years (under-5) deaths per 1000 live births,^5^ accounts for 14% of the global burden of under-5 mortality.^3,6^ Meeting the global child mortality SDG target is therefore intrinsically tied to India’s success.

Targeting reduction in mortality risk for children younger than 5 years requires a disaggregation by age. However, deaths during the first 7 days (early neonatal) and 8 to 28 days (late neonatal) are usually combined. Similarly, deaths occurring between the ages of 1 to 11 months (postneonatal) and 12 to 59 months (child) are also often conflated. This is problematic as the causes of death during different ages are distinct, necessitating different interventions at each stage of life.^7,8^ During the early-neonatal period, most deaths occur due to preterm birth complications or intrapartum-related events,^7,8^ whereas malnutrition and infections are the major causes of death during late-neonatal, postneonatal, or childhood periods.^7,9,10^ The mortality risk also varies across ages during early years.^11^

To our knowledge, systematic assessments of how the patterns of early-neonatal, late-neonatal, postneonatal, and child mortality have changed over time in India and at subnational levels, especially accounting for the changing geographic boundaries of Indian states have not been conducted (eMethods 1 in Supplement 1). For India to develop a successful child survival policy framework, a detailed assessment of mortality patterns over time is essential. We present a disaggregated and up-to-date assessment of changes in mortality risk and percentage share of burden for each age period to total deaths in the under-5 population in India and across its 36 states and union territories (UTs) from 1993 to 2021. We also assess the progress states and UTs are making toward achieving SDG targets for early-neonatal, late-neonatal, postneonatal, and child mortality.

## Methods

### Study Design

This study followed the Strengthening the Reporting of Observational Studies in Epidemiology (STROBE) reporting guideline for cross-sectional studies. We used 5 waves of the National Family Health Surveys (NFHS), conducted in 1992-1993,^12^ 1998-1999,^13^ 2005-2006,^14^ 2015-2016,^15^ and 2019-2021^5^; hereafter, identified with the end year of each survey, although the reference date for the estimated 5-year rates is approximately 2½ years before the mean date of the interview for each survey. The mortality data are representative nationally, as well as at the level of states and UTs. The NFHS follows the protocol and procedures of the global Demographic and Health Surveys (DHS) Program currently active in more than 90 countries.^16,17^ We restricted our analysis to the NFHS as the data source to maintain comparability in the method of data collection for mortality and because the NFHS provides micro data with geographic identifiers below the level of states. This was necessary as the geometry and number of states and UTs changed over the survey years, with the latest configuration being 28 states and 8 UTs. To make states and UTs comparable over time, we adopted a published method that entailed reassigning district-level information from older surveys to states according to the most recent geometry.^18^ The NFHS data were collected using informed consent and the protocol for the survey, including the content of all the questionnaires, was approved by the International Institute for Population Studies Institutional Review Board (IRB) and the ICF IRB. For the analysis presented in this study using the NFHS data, the Harvard Longwood Campus IRB allows researchers to self-determine whether their research meets the requirements of IRB oversight using the IRB Decision Tool. These activities did not meet the regulatory definition of human participant research, and our study was determined to be exempt from a full institutional review.

### Study Population

The study population was children who were born in the 5 years preceding each of the surveys. Detailed information on each birth was collected from the mother or primary caretaker. Observations for which the date of birth or age at death of the child were missing or unknown were imputed following DHS imputation procedures.^19^

### Outcomes

The surveys recorded the month and year the child was born, whether the child was still alive, and if not alive, the age of death in days if less than age 1 month, in months if less than 2 years, and in years if older than 2 years. The underlying sample data are provided in eTables 8-12 in Supplement 1.

Using these data, we computed the following mortality rates: early-neonatal mortality rate (ENMR) (the number of deaths occurring in the first 7 completed days after the child is born per 1000 live births, late-neonatal mortality rate (LNMR) (the number of deaths occurring on days 8 to 28 completed days after the child is born per 1000 live births, postneonatal mortality rate (PNMR) (the number of deaths occurring on days 29 to 11 completed months per 1000 children who are at least 29 days old, and childhood mortality rate (CMR) (the number of deaths occurring in months 12 to 59 completed months per 1000 children who are at least aged 1 year) (eMethods 1 in Supplement 1).^20^ Hereafter, all age-specific mortalities are referred to as *deaths per 1000*.

The proportion of children who died (probability of death) from the cohort of children included at the starting age of the age bracket was then computed. We used real birth cohorts for the ENMR, LNMR, and overall neonatal mortality rates and the synthetic cohort lifetable approach to calculate the infant and child mortality rates for each of the age groups, as implemented by the DHS.^21^ Postneonatal mortality is calculated as infant mortality (calculated via synthetic cohort) minus neonatal mortality (calculated via real cohort). Mortality information collected for a year includes age-specific deaths from more than 1 cohort for a given time.^22^ The synthetic cohort approach combines the period spent by children of all cohorts in each survey. The probability of death was then calculated separately for the following age intervals: less than 1 month, 1 to 2 months, 3 to 5 months, 6 to 11 months, 12 to 23 months, 24 to 35 months, 36 to 47 months, and 48 to 59 months. The age-specific probabilities of dying in the specified period were combined to derive early-neonatal, late-neonatal, postneonatal, and child mortality rates and computed for each survey year for all-India and 36 states and UTs. The Stata codes to estimate the mortality rates are provided in eMethods 3 in Supplement 1. We visualized the changing geographic distribution of ENMR, LNMR, PNMR, and CMR using choropleth maps and also created an online interactive dashboard (eFigure 2 in Supplement 1).

### Statistical Analysis

#### Quantifying Change Across Years

The number of years between the surveys was not the same. For instance, there was a 10-year interval between the 2006 and 2016 surveys, but a 5-year interval between the 2016 and 2021 surveys. We compared the absolute change in mortality rates between different time periods using standardized absolute change (SAC) defined as:

SAC = ([*P_t_*−*P_t–n_*]/*n*),where *P_t_* is the mortality rate for the time *t*, *P_t − n_* is mortality rate *n* years before *t*, and *n* represents the number of years between any 2 surveys (taking the latest survey year of multiyear surveys). A negative SAC value indicates a decrease in mortality, whereas a positive SAC value indicates an increase. We computed the Pearson correlation coefficient between each of the 4 mortality rates for 1993 and 2021 (eTable 5 in Supplement 1).^23^

#### Estimating the Share of Mortality Burden

Since each mortality rate is exclusive to the 4 age groups with no overlaps, we added the 4 mortality rates to obtain an adjusted under-5 mortality rate. Each of the age-specific rates was then divided by the calculated under-5 mortality rate and multiplied by 100 to obtain the percentage share of early-neonatal, late-neonatal, postneonatal, and child deaths to total under-5 deaths.

#### Estimating Progress on SDGs

We used a published method to estimate progress toward the SDG targets for each state and UT.^24^ This method assumes that the SAC between 2016 and 2021 is maintained until 2030 and classifies each state and UT into 1 of 4 categories for each outcome: achieved-I (goal already met in 2021 and will continue to be met in 2030), achieved-II (goal already met in 2021 but will no longer maintain status by 2030), on-target (goal not met in 2021 but will be met by 2030), and off-target (goal not met in 2021 and will not be met by 2030). The SDG only sets targets for NMR (12 deaths per 1000 live births) and under-5 mortality rate (25 deaths per 1000 live births).^2^ The targets used in this study for the different age-periods are, therefore, approximations and meant to provide a general sense of the progress for a particular geographic unit. Given the neonatal and under-5 mortality targets, the target for postneonatal and child mortality rates combined is approximately 13 deaths per 1000. Consequently, we chose the postneonatal mortality target as 8 deaths per 1000, the child mortality target as 5 deaths per 1000, the early-neonatal target as 7 deaths per 1000 live births, and the late-neonatal target as 5 deaths per 1000. Analyses were conducted used Stata, version 17 (StataCorp LLC).^23^

## Results

The final weighted analytical sample included 6879 (1993), 5482 (1999), 4293 (2006), 12612 (2016), and 9788 (2021) children who died before their fifth birthday in the past 5 years of each survey (eTables 8-12 in Supplement 1). Specifically, the weighted sample included 2114 (1993), 1810 (1999), 1710 (2006), 6221 (2016), and 4836 (2021) early-neonatal deaths, 881 (1993), 621 (1999), 492 (2006), 1224 (2016), and 978 (2021) late-neonatal deaths, 1895 (1993), 1403 (1999), 1048 (2006), 2858 (2016), and 2346 (2021) postneonatal deaths, and 1989 (1993), 1648 (1999), 1043 (2006), 2309 (2016), and 1628 (2021) child deaths (eTables 8-12 in Supplement 1).

**Figure 1.**
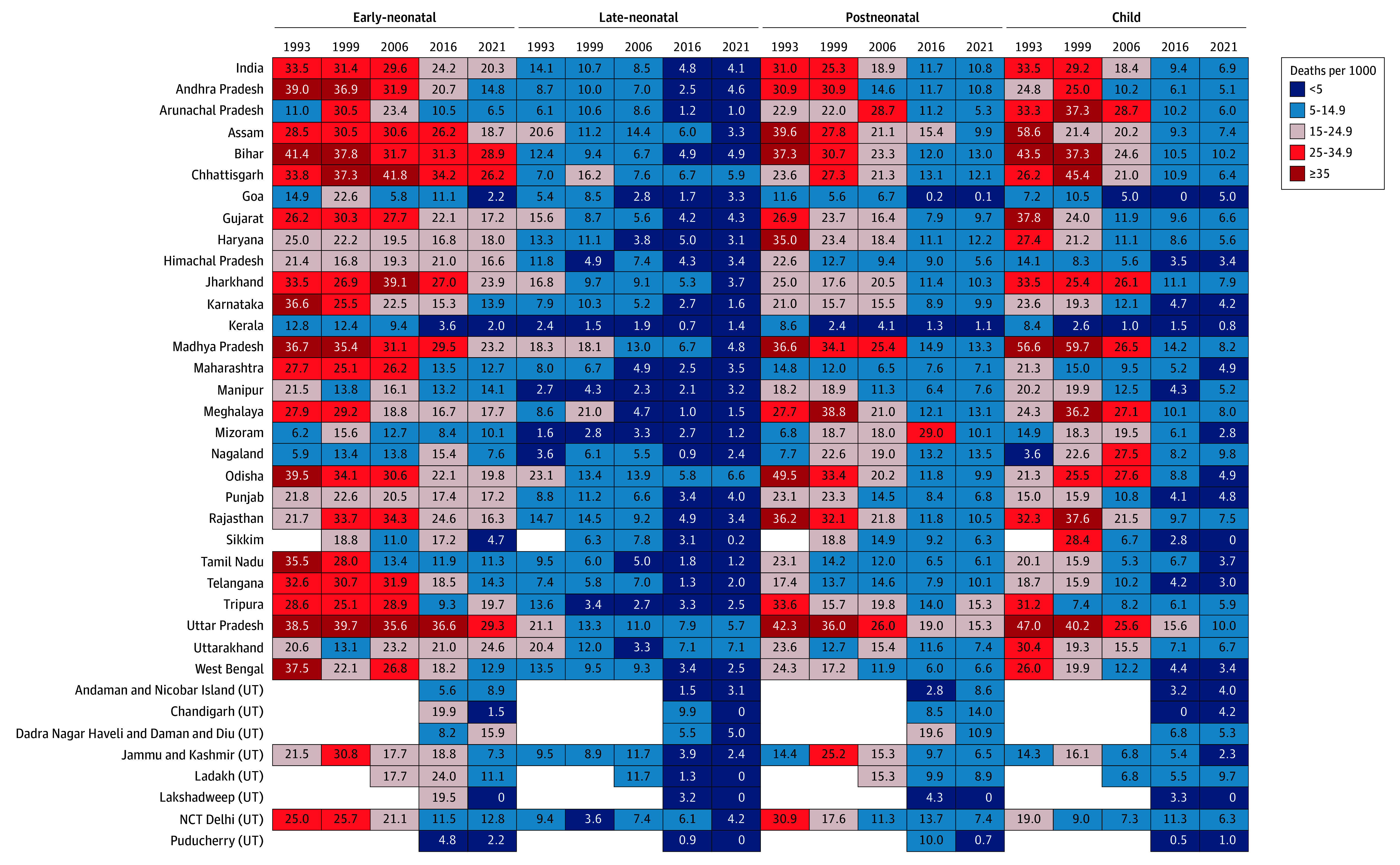
Early-Neonatal, Late-Neonatal, Postneonatal, and Child Mortality Rates for States and Union Territories (UT) of India, 1993-2021 The collective term *deaths per 1000* encompasses all mortalities (early and late neonatal periods, *deaths per 1000 live births*; postneonatal, *deaths per 1000 children aged at least 29 days;* and child, *deaths per 1000 children aged at least 1 year*). NCT Delhi indicates National Capital Territory of Delhi.

In 2021, mortality risk was highest for ENMR followed by PNMR and CMR, with the lowest risk being the late-neonatal period (eTables 1-4 in Supplement 1). However, in 1993, the mortality risk was the highest for both the ENMR and CMR at 33.5 deaths per 1000 followed by PNMR and LNMR (eTables 1-4 in Supplement 1). Between 1993 and 2021, India observed a substantial decrease in all 4 mortality rates (Figure 1; eTables 1-4 in Supplement 1). The largest decrease (in absolute percentage points) was observed for CMR followed by PNMR, ENMR, and LNMR. The CMR decreased from 33.5 (95% CI, 31.5-35.5) to 6.9 (95% CI, 6.5-7.4), and the PNMR decreased from 31.0 (95% CI, 31.0-31.0) to 10.8 (95% CI, 10.8-10.8). Meanwhile, the LNMR decreased from 14.1 (95% CI, 13.8-14.5) to 4.1 (95% CI, 3.7-4.4), and the ENMR decreased from 33.5 (95% CI, 32.9-34.1) to 20.3 (95% CI, 19.6-21.1).

**Figure 2.**
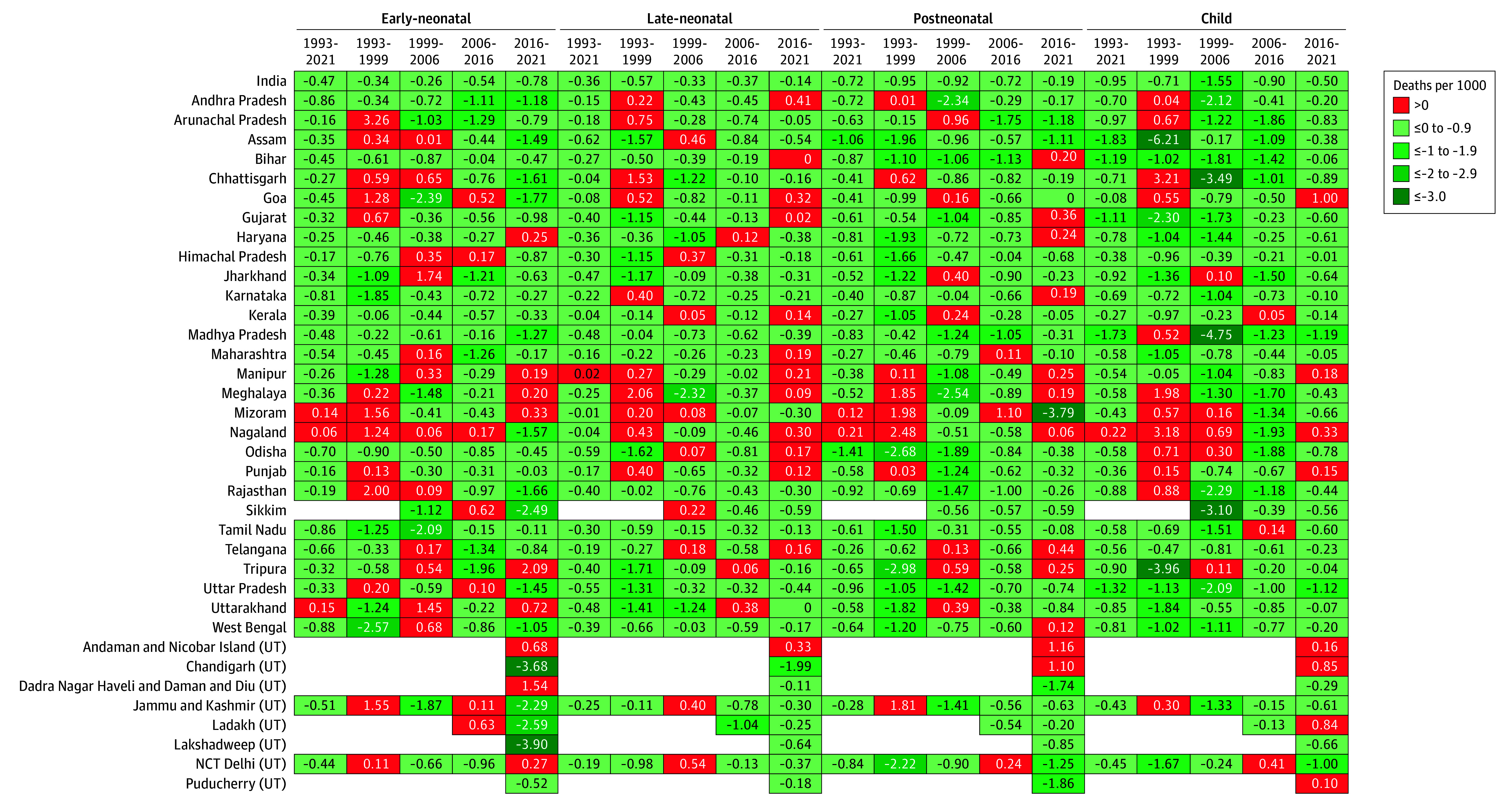
Standardized Absolute Change for Early-Neonatal, Late-Neonatal, Postneonatal and Child Mortality Rates for States and Union Territories (UT) of India Between 1993-2021, 1993-1999, 1999-2006, 2006-2016, and 2016-2021 The collective term *deaths per 1000* encompasses all mortalities (early and late neonatal periods, *deaths per 1000 live births*; postneonatal, *deaths per 1000 children aged at least 29 days;* and child, *deaths per 1000 children aged at least 1 year*). NCT Delhi indicates National Capital Territory of Delhi. Red cells indicate an increase in the mortality rate for that specific time period.

There were considerable differences in the mortality decrease patterns across the different time periods and age groups. For the ENMR, the period of the greatest average annual decrease was 2016-2021 (SAC = −0.78); the greatest average annual decrease was 1993-1999 (SAC = −0.57) for the LNMR, 1993-1999 (SAC = −0.95) for the PNMR, and 1999-2006 (SAC = −1.55) for the CMR (Figure 2). The least reductions in average annual decrease for the ENMR were observed between 1999 and 2006; for the LNMR, PNMR, and CMR it was 2016-2021.

### Changes in Mortality Rates Across States and UTs

Except for Nagaland, Mizoram, Uttarakhand, and Manipur, all 4 mortality rates decreased in the rest of the states and UTs between 1993 and 2021 (Figures 1 and 2). During this period, Nagaland experienced a worsening of ENMR (SAC = 0.06), PNMR (SAC = 0.21), and CMR (SAC = 0.22), whereas Mizoram saw a worsening of ENMR (SAC = 0.14) and PNMR (SAC = 0.12). Uttarakhand saw a worsening for ENMR (SAC = 0.15), whereas Manipur saw worsening of LNMR (SAC = 0.02). There was considerable variation in the amount of reduction experienced across states and UTs (Figure 2).

While most of the states and UTs have seen some decrease in all 4 mortality rates, some concerning patterns in the most recent time periods were observed. Between 2016 and 2021, early-neonatal mortality increased in 9 states and UTs, late-neonatal mortality increased in 13 states and UTs, postneonatal mortality increased in 12 states and UTs, and child mortality increased in 8 states and UTs. Meanwhile, from 2006 to 2016, 7 states and UTs experienced an increase in early-neonatal mortality, and 3 states and UTs for late-neonatal mortality, postneonatal mortality, and child mortality.

**Figure 3.**
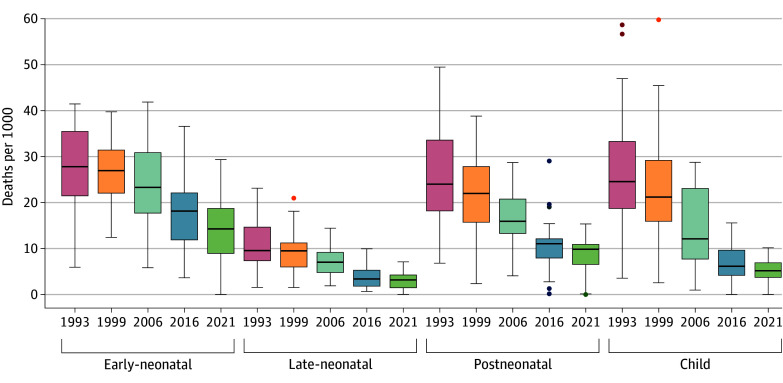
Summary Distribution of Early-Neonatal, Late-Neonatal, Postneonatal, and Child Mortality Rates Across States and Union Territories of India, 1993-2021 The collective term *deaths per 1000* encompasses all mortalities (early and late neonatal periods, *deaths per 1000 live births*; postneonatal, *deaths per 1000 children aged at least 29 days;* and child, *deaths per 1000 children aged at least 1 year*). The horizontal bar inside the box indicates the median, the lower and upper ends of the boxes are the 25th and 75th percentile respectively and represent the interquartile range (IQR). The whiskers indicate data 1.5 times the IQR and the circles indicate outliers.

The interstate inequalities in all 4 mortality rates were reduced (Figure 3), with CMR experiencing the largest reduction (summary distribution, 13.2-2.7), followed by PNMR (summary distribution, 10.6-4.0), LNMR (summary distribution, 5.9-1.9), and ENMR (summary distribution, 9.9-7.7) (eTable 6 in Supplement 1). On average, states with higher baseline mortality rates in 1993 experienced the largest decrease between 1993 and 2021 (eFigure 1, eTable 6 in Supplement 1). While there was some degree of variation, overall, there was a moderate to strong correlation between the 4 mortality rates across states (eTable 5 in Supplement 1).

### Changes in Share of Mortality Burden for Different Ages Across States and UTs

**Figure 4.**
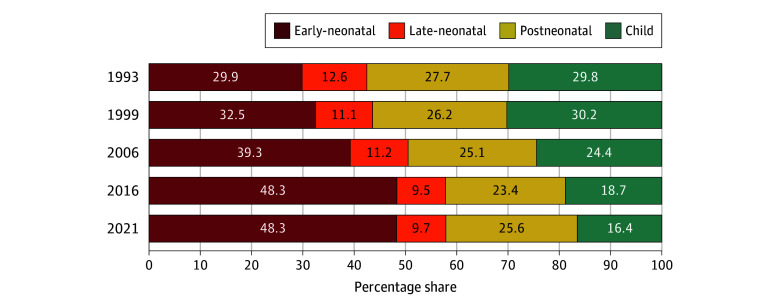
Percentage Share of the Burden of Early-Neonatal, Late-Neonatal, Postneonatal, and Child Mortality to Under-5 Mortality for India, 1993-2021

The share of early-neonatal deaths to total under-5 deaths increased from 29.9% in 1993 to 48.3% in 2021, while the share of late-neonatal deaths decreased from 12.6% in 1993 to 9.7% in 2021, postneonatal deaths from 27.7% in 1993 to 25.6% in 2021, and child deaths from 29.8% in 1993 to 16.4% in 2021 (Figure 4). Except for Kerala, Goa, and Nagaland, the remaining states and UTs with available data for both time periods experienced an increase in the share of early-neonatal deaths. Substantial shifting of the share was observed between postneonatal and child deaths between 1993 and 2021 (eTable 7 in Supplement 1). In 2016, nearly 50% of all deaths in the under-5 population in India occurred within 7 days after a child's birth. This has remained unchanged in 2021.

### Progress Toward SDG Targets for Age-Specific Mortalities

**Figure 5.**
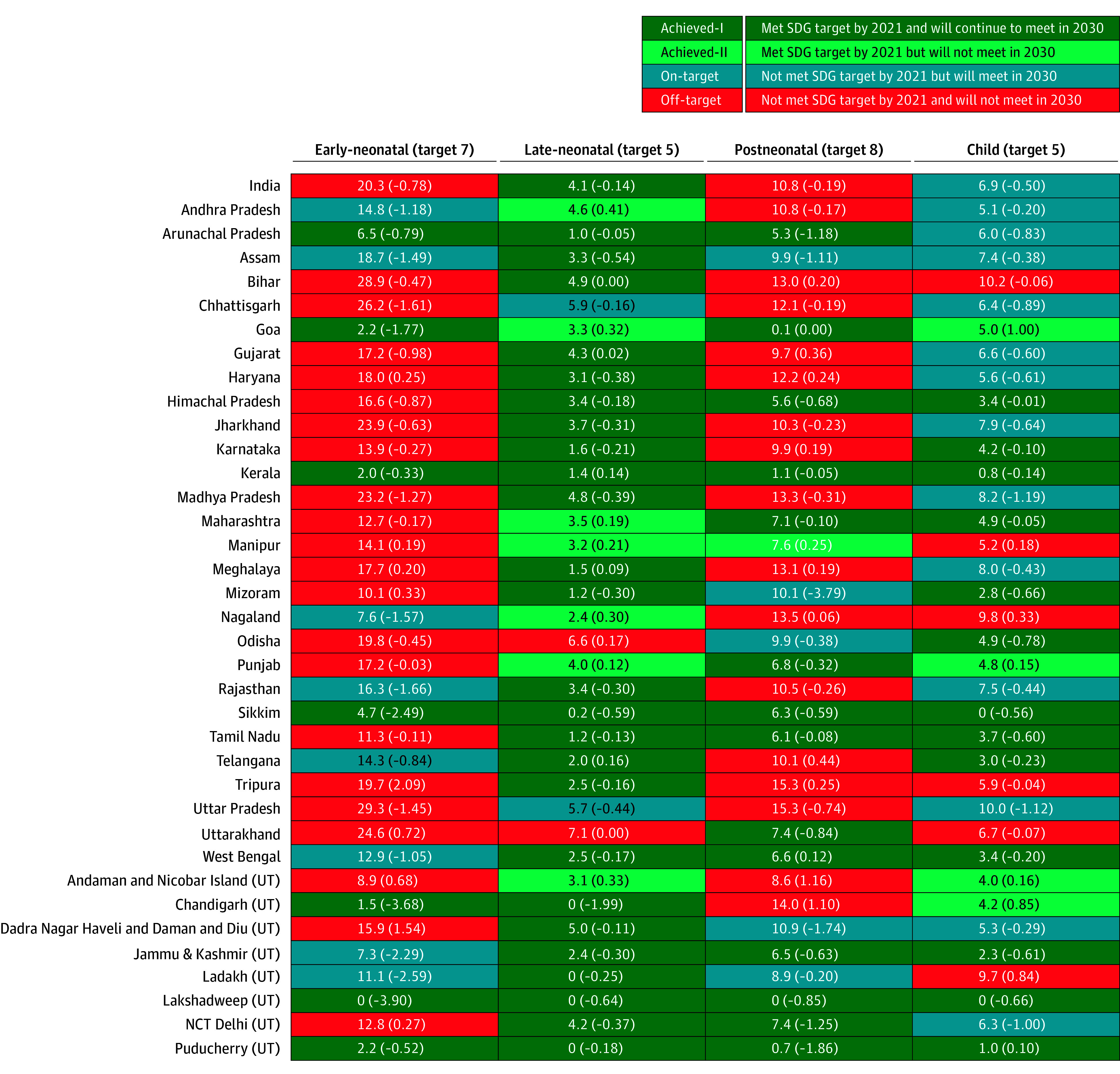
State and Union Territory (UT) Distribution of Sustainable Development Goal (SDG) Status for Neonatal, Postneonatal, and Child Mortality, 2021 NCT Delhi indicates National Capital Territory of Delhi.

Based on the most recent rate of change, from 2016 to 2021, India will not meet the SDG targets for early-neonatal and postneonatal mortality by 2030 (Figure 5). Twenty-one states and UTs for ENMR, 9 for LNMR, 17 for PNMR, and 10 for CMR will fail to meet their SDG targets by 2030. Fourteen states and UTs for PNMR, 25 for LNMR, 13 for CMR, and 7 for ENMR have met their SDG targets and will continue to meet the targets in 2030.

## Discussion

Our study has 4 salient findings. First, mortality rates in the late-neonatal and child phases are the lowest and have decreased markedly over the years, along with a substantial reduction in interstate inequalities. Second, the mortality rate is highest within the first week (early-neonatal phase), followed by the postneonatal phase, and interstate inequalities also remain notable. Third, the share of mortality burden is now concentrated during the early-neonatal and postneonatal periods, underscoring the importance of the first week and first year of life, past the late-neonatal period. Fourth, India overall, along with a considerable number of states and UTs, will not meet the SDG targets related to child survival during early-neonatal and postneonatal periods. We elaborate on these findings with an emphasis on the early-neonatal and postneonatal phases since mortality risk and burden during these 2 periods remain high.

Child survival in the first week of life is mostly centered around quality of child delivery settings, newborn care, and conditions related to the child and the mother.^8^ While India has made substantial advances in increasing the percentage of institutional deliveries (from 26% in 1993 to 89% in 2021^5,12^), translating these increases to a reduction in early-neonatal mortality has not occurred at the same pace. It is critical that, as India develops a strategy for further reduction in ENMRs, the focus is shifted from the quantity toward the quality of the institutions where delivery and newborn care occur.^25^ Risk factors of early-neonatal mortality, such as a higher prevalence of low birth weight,^5^ and congenital malformations, are also highly prevalent in India.^26^ With identification of high-risk pregnancies in a timely manner still being less than optimal,^27^ maternal mortality during delivery also remains high in India (at 99 per 100 000 live births in 2020^28^), which is likely to also increase the early-neonatal mortality rate. Given these conditions, there is an immediate need to focus on the quality aspect of institutional deliveries, including assessing and building neonatal intensive care units to meet the SDG targets related to early-life mortality.

It is noteworthy that the mortality rate during the late-neonatal phase (deaths in 8-28 days) significantly diminished in comparison with the early-neonatal phase, and then increased during the postneonatal phase (deaths in 29 days to 11 completed months). Even though the underlying biological risk is expected to decrease with age systematically for children younger than 5 years, the increase during the postneonatal phase, with the rate being more than twice that of late-neonatal phase, merits further research.

Existing research on the high prevalence of food deprivation among young children in India suggests that the mortality burden during the postneonatal period could be substantial.^18^ For instance, about 30% of children aged 6 to 12 months (ie, the latter half of the postneonatal period) were reported to have not consumed any food for the 24 hours before the survey.^18^ Exclusive breastfeeding (which is only recommended for the first 6 months after birth) remains highly prevalent in India even after 6 months, thus depriving the child of the crucial nutritional requirements.^29^ Even with substantial progress in expansion of recommended vaccination coverage among young infants as well as overall improvements in environmental conditions, such as sanitation, and thereby reducing susceptibility to environmental infections, the decrease in postneonatal mortality rates over time has not been as substantial as observed for child mortality rates. While there have been marked improvements in expanding vaccination coverage across India, a substantial number of children do not receive any routine vaccinations.^30^ Furthermore, preliminary evidence suggests that the impact of COVID-19 may also have adversely affected the continued expansion of the vaccination.^31^ Going forward, India needs to closely examine its existing policies that are directly or indirectly related to child survival (eMethods 2 in Supplement 1) and develop a specific strategy focused on the postneonatal period, including further examination on what periods during this time matter the most, along with a concerted effort to eliminate any form of food deprivation. The government of India has approved a resolution to provide free food grains to more than 813 million people in India through the Pradhan Mantri Garib Kalyan Anna Yojana program starting January 1, 2024, for a period of 5 years,^32^ which if implemented effectively can help with reducing the mortality burden during the postneonatal period.

### Limitations

Our findings should be interpreted alongside the following data-related considerations. First, our estimates rely on maternal reports of birth dates and age at death, thus introducing potential recall bias. While bias is noted for death reporting beyond 5 years, the 0- to 4-year recall period used in our study largely remains unbiased.^33^ Second, although NFHS coverage improved, data for several UTs were only available for 2016 and 2021. These include Lakshadweep, Puducherry, Chandigarh, Andaman and Nicobar, Dadra and Nagar Haveli, and Daman and Diu. However, these areas constitute less than 1% of India’s population.^34^ Third, due to 2006 survey limitations, we assume that the estimates of new state divisions are the same as their parent state (Ladakh and Telangana). Finally, given the arbitrariness of the definition of the WHO age-specific periods, there is a need for further research to more precisely understand the association between age and mortality risk occurring during childhood years, especially during the first 2 years. While these data considerations need acknowledgment, they do not affect the overall robustness of the findings related to understanding the evolution of mortality among neonates, postneonates, and children in India.

## Conclusion

In this repeated cross-sectional study, we provided an up-to-date assessment of age-specific mortality during the first 5 years of a child’s life and how this has evolved in India over the past 30 years. Even as mortality rates have seen a remarkable decrease over the last 30 years, there is crucial need to focus on the early-neonatal and postneonatal periods. Our findings also reveal persistent inequalities across states and UTs, especially for early-neonatal and postneonatal mortality rates, with recent years showing a stagnation or worsening in certain states and UTs. It is critical for policy makers to focus on the specific states and UTs that are of concern and develop context-specific interventions. This is critical to ensure that India attains the SDG targets related to mortality during early life years and, in doing so, positively contribute to the global progress on child survival.

## Notes

Supplement 1. **eTable 1**. Early-Neonatal Mortality Rate and 95% Confidence Intervals for States/Union Territories of India, 1993-2021 **eTable 2**. Late-Neonatal Mortality Rate and 95% Confidence Intervals for States/Union Territories of India, 1993-2021 **eTable 3**. Postneonatal Mortality Rate and 95% Confidence Intervals for States/Union Territories of India, 1993-2021 **eTable 4. **Child Mortality Rate and 95% Confidence Intervals for States/Union Territories of India, 1993-2021 **eTable 5.** Pearson Correlation Coefficient between Early-Neonatal, Late-Neonatal, Postneonatal and Child Mortality Rates Across States/Union Territories, 1993 and 2021 **eTable 6. **Standard Deviation (SD) and Interquartile Range (IQR) of Early-Neonatal, Late-Neonatal, Postneonatal and Child Mortality Rates of States/Union Territories of India, 1993-2021 **eTable 7. **Percentage Share of the Burden of Early-Neonatal, Late-Neonatal, Postneonatal and Child Mortality to Under 5 Mortality Across States/Union Territories of India, 1993-2021 **eTable 8. **Distribution of the Observed Weighted Sample of Early-Neonatal, Late-Neonatal, Postneonatal, and Child Deaths Across All States/Union Territories, 1993 **eTable 9. **Distribution of the Observed Weighted Sample of Early-Neonatal, Late-Neonatal, Postneonatal, and Child Deaths Across All States/Union Territories, 1999 **eTable 10. **Distribution of the Observed Weighted Sample of Early-Neonatal, Late-Neonatal, Postneonatal, and Child Deaths Across All States/Union Territories, 2006 **eTable 11. **Distribution of the Observed Weighted Sample of Early-Neonatal, Late-Neonatal, Postneonatal, and Child Deaths Across All States/Union Territories, 2016 **eTable 12. **Distribution of the Observed Weighted Sample of Early-Neonatal, Late-Neonatal, Postneonatal, and Child Deaths Across All States/Union Territories, 2021 **eFigure 1.** Relationship Between Standardized Absolute Change (1993-2021) and Baseline Early-Neonatal, Late-Neonatal, Postneonatal and Child Mortality Rates (1993) Across States/Union Territories of India **eFigure 2.** Interactive Dashboard Showing Geographic Distribution of Early-Neonatal, Late-Neonatal, Postneonatal and Child Mortality Rates Across the States and Union Territories of India, 1993-2021 **eMethods 1**. Systematic Search of Prior Literature
eReferences
**eMethods 2.** National Policies That May Directly or Indirectly Influence Childhood Mortality in India **eMethods 3. **Stata Codes to Estimate Early-Neonatal, Late-Neonatal, Postneonatal and Child Mortality Rates Across States/Union Territories of India, 1993 to 2021

Supplement 2.
Data Sharing Statement

## References

[1] United Nations Development Programme. The SDGs in action. Accessed February 2024. https://www.undp.org/sustainable-development-goals?gclid=EAIaIQobChMIjvHb1cTRgQMVCaVmAh29iAxyEAAYAyAAEgI5avD_BwE

[2] United Nations Department of Economic and Social Affairs. SDG 3 Ensure healthy lives and promote well-being for all at all ages. Accessed February 2024. https://sdgs.un.org/goals/goal3

[3] Levels & Trends in Child Mortality. Report 2022, Estimates developed by the United Nations Inter-agency Group for Child Mortality Estimation. 2023. Accessed February 2024. https://childmortality.org/wp-content/uploads/2023/01/UN-IGME-Child-Mortality-Report-2022.pdf

[4] Sharrow D, Hug L, You D, ; UN Inter-agency Group for Child Mortality Estimation and its Technical Advisory Group. Global, regional, and national trends in under-5 mortality between 1990 and 2019 with scenario-based projections until 2030: a systematic analysis by the UN Inter-agency Group for Child Mortality Estimation. Lancet Glob Health. 2022;10(2):e195-e206. doi:10.1016/S2214-109X(21)00515-5 35063111 PMC8789561

[5] International Institute for Population Sciences. *National Family Health Survey (NFHS-5), 2019-21*. March 2022. Accessed April 1, 2024. https://dhsprogram.com/pubs/pdf/FR375/FR375.pdf

[6] World Population Prospects. 2022. United Nations, Department of Economic and Social Affairs, Population Division. Accessed February 2024. https://population.un.org/wpp/Download/Standard/Mortality/

[7] Perin J, Mulick A, Yeung D, . Global, regional, and national causes of under-5 mortality in 2000-19: an updated systematic analysis with implications for the Sustainable Development Goals. Lancet Child Adolesc Health. 2022;6(2):106-115. doi:10.1016/S2352-4642(21)00311-4 34800370 PMC8786667

[8] Oza S, Lawn JE, Hogan DR, Mathers C, Cousens SN. Neonatal cause-of-death estimates for the early and late neonatal periods for 194 countries: 2000-2013. Bull World Health Organ. 2015;93(1):19-28. doi:10.2471/BLT.14.139790 25558104 PMC4271684

[9] Bassat Q, Blau DM, Ogbuanu IU, ; Child Health and Mortality Prevention Surveillance (CHAMPS) Network. Causes of death among infants and children in the Child Health and Mortality Prevention Surveillance (CHAMPS) network. JAMA Netw Open. 2023;6(7):e2322494-e2322494. doi:10.1001/jamanetworkopen.2023.22494 37494044 PMC10372710

[10] Subramanian SV. Need for a structural approach to promote child survival. JAMA Netw Open. 2023;6(7):e2322435-e2322435. doi:10.1001/jamanetworkopen.2023.22435 37494048

[11] Karlsson O, Kim R, Hasman A, Subramanian SV. Age distribution of all-cause mortality among children younger than 5 years in low-and middle-income countries. JAMA Netw Open. 2022;5(5):e2212692-e2212692. doi:10.1001/jamanetworkopen.2022.12692 35587349 PMC9121187

[12] International Institute for Population Sciences. National Family Health Survey: MCH and Family Planning, India 1992-93. International Institute for Population Sciences; 1995.

[13] International Institute for Population Sciences. National Family Health Survey (NFHS-2), 1998-99. International Institute for Population Sciences; 2000.

[14] International Institute for Population Sciences. National Family Health Survey (NFHS-3), 2005-06. International Institute for Population Sciences; 2007.

[15] International Institute for Population Sciences. *National Family Health Survey (NFHS-4), 2015-16*. Accessed April 1, 2024. https://dhsprogram.com/pubs/pdf/FR339/FR339.pdf 12290290

[16] Corsi DJ, Neuman M, Finlay JE, Subramanian SV. Demographic and health surveys: a profile. Int J Epidemiol. 2012;41(6):1602-1613. doi:10.1093/ije/dys184 23148108

[17] USAID. The DHS Program. Accessed February 2024. https://dhsprogram.com/

[18] Subramanian SV, Ambade M, Sharma S, Kumar A, Kim R. Prevalence of Zero-Food among infants and young children in India: patterns of change across the States and Union Territories of India, 1993-2021. EClinicalMedicine. 2023;58:101890. doi:10.1016/j.eclinm.2023.101890 37065175 PMC10102207

[19] Croft T. DHS data editing and imputation. Accessed February 2024. https://dhsprogram.com/pubs/pdf/DHSG3/DHS_Data_Editing.pdf

[20] The DHS Program User Forum. Accessed February 2024. https://userforum.dhsprogram.com/index.php?t=msg&goto=13420&S=Google

[21] Croft TN, Marshall AM, Allen CK, Arnold F, Assaf S, Balian S. Guide to DHS Statistics. ICF; 2018:645.

[22] Moultrie TA, Dorrington RE, Hill AG, Hill K, Timæus IM, Zaba B. Tools for Demographic Estimation. International Union for the Scientific Study of Population; 2013.

[23] StataCorp. 2023. Stata statistical software: release 17. StataCorp LLC. Accessed April 22, 2024. https://www.stata.com/

[24] Subramanian SV, Ambade M, Kumar A, . Progress on Sustainable Development Goal indicators in 707 districts of India: a quantitative mid-line assessment using the National Family Health Surveys, 2016 and 2021. Lancet Reg Health Southeast Asia. 2023;13:100155. doi:10.1016/j.lansea.2023.100155 37383562 PMC10306006

[25] Kruk ME, Gage AD, Arsenault C, . High-quality health systems in the Sustainable Development Goals era: time for a revolution. Lancet Glob Health. 2018;6(11):e1196-e1252. doi:10.1016/S2214-109X(18)30386-3 30196093 PMC7734391

[26] Bhide P, Kar A. A national estimate of the birth prevalence of congenital anomalies in India: systematic review and meta-analysis. BMC Pediatr. 2018;18(1):175. doi:10.1186/s12887-018-1149-0 29801440 PMC5970488

[27] Kapoor M, Kim R, Sahoo T, . Association of maternal history of neonatal death with subsequent neonatal death in India. JAMA Netw Open. 2020;3(4):e202887-e202887. doi:10.1001/jamanetworkopen.2020.2887 32297947 PMC7163408

[28] Meh C, Sharma A, Ram U, . Trends in maternal mortality in India over two decades in nationally representative surveys. BJOG. 2022;129(4):550-561. doi:10.1111/1471-0528.16888 34455679 PMC9292773

[29] Chen Z, Sharma S, Chen S, Kim R, Subramanian SV, Li Z. Prevalence, trend, and inequality of prolonged exclusive breastfeeding among children aged 6-23 months old in India from 1992-2021: a cross-sectional study of nationally representative, individual-level data. J Glob Health. 2024;14:04026. doi:10.7189/jogh.14.04026 38334279 PMC10854209

[30] Rajpal S, Kumar A, Johri M, Kim R, Subramanian SV. Patterns in the prevalence of unvaccinated children across 36 states and union territories in India, 1993-2021. JAMA Netw Open. 2023;6(2):e2254919-e2254919. doi:10.1001/jamanetworkopen.2022.54919 36763362 PMC9918883

[31] Ko S, Kim R, Subramanian SV. Patterns in child health outcomes before and after the COVID-19 outbreak in India. JAMA Netw Open. 2023;6(6):e2317055-e2317055. doi:10.1001/jamanetworkopen.2023.17055 37273207 PMC10242422

[32] Free Foodgrains for 81.35 crore beneficiaries for five years: cabinet decision. 2023. Accessed February 2024. https://pib.gov.in/PressReleasePage.aspx?PRID=1980686

[33] Neal S. The measurement of neonatal mortality: how reliable is demographic and household survey data? ESRC Centre for Population Change Working Paper Series. 2012;25.

[34] Office of the Registrar General & Census Commissioner I. Ministry of Home Affairs, Government of India. Census tables. Accessed February 2024. https://censusindia.gov.in/census.website/data/census-tables

