# Errors in Abstract, Results, Supplement, and Figure 4

**DOI:** 10.1001/jamanetworkopen.2025.16129

**Published:** 2025-05-19

In the Original Investigation titled, “Early-Neonatal, Late-Neonatal, Postneonatal, and Child Mortality Rates Across India, 1993-2021”^1^ published May 10, 2024, there was an error in the Abstract, Results and Supplementary file that incorrectly reported the number of sample deaths in each NFHS survey. The numbers originally reported represent the under five deaths from the complete birth history from each mother rather than only from the five years that precede each mother’s interview. This error does not affect any of the mortality rates that were presented in the original paper as those were correctly calculated based on under five deaths that occurred five years before each mother’s interview and therefore do not affect the overall findings, interpretations, or conclusions of the article. The correct number of deaths included in the final analytical sample are 2114 (1993), 1810 (1999), 1710 (2006), 6221 (2016), and 4836 (2021) early-neonatal deaths, 881 (1993), 621 (1999), 492 (2006), 1224 (2016), and 978 (2021) late-neonatal deaths, 1895 (1993), 1403 (1999), 1048 (2006), 2858 (2016), and 2346 (2021) postneonatal deaths, and 1989 (1993), 1648 (1999), 1043 (2006), 2309 (2016), and 1628 (2021) child deaths (eTables 8-12 in Supplement 1). In the x-axis of Figure 4, the spelling error has been corrected to “Percentage.” The authors have explained what happened in a Comment,^2^ and this article has been corrected with updated Supplement materials.^1^
