# Supplementary material for: Early-Neonatal, Late-Neonatal, Postneonatal, and Child Mortality Rates Across India, 1993-2021
Source: JAMA Netw Open. 2024 May 10;7(5):e2410046. doi: 10.1001/jamanetworkopen.2024.10046 (PMC11087840; doi:10.1001/jamanetworkopen.2024.10046)
Supplement: Supplement 1. — eTable 1. Early-Neonatal Mortality Rate and 95% Confidence Intervals for States/Union Territories of India, 1993-2021 eTable 2. Late-Neonatal Mortality Rate and 95% Confidence Intervals for States/Union Territories of India, 1993-2021 eTable 3. Postneonatal Mortality Rate and 95% Confidence Intervals for States/Union Territories of India, 1993-2021 eTable 4. Child Mortality Rate and 95% Confidence Intervals for States/Union Territories of India, 1993-2021 eTable 5. Pearson Correlation Coefficient between Early-Neonatal, Late-Neonatal, Postneonatal and Child Mortality Rates Across States/Union Territories, 1993 and 2021 eTable 6. Standard Deviation (SD) and Interquartile Range (IQR) of Early-Neonatal, Late-Neonatal, Postneonatal and Child Mortality Rates of States/Union Territories of India, 1993-2021 eTable 7. Percentage Share of the Burden of Early-Neonatal, Late-Neonatal, Postneonatal and Child Mortality to Under 5 Mortality Across States/Union Territories of India, 1993-2021 eTable 8. Distribution of the Observed Weighted Sample of Early-Neonatal, Late-Neonatal, Postneonatal, and Child Deaths Across All States/Union Territories, 1993 eTable 9. Distribution of the Observed Weighted Sample of Early-Neonatal, Late-Neonatal, Postneonatal, and Child Deaths Across All States/Union Territories, 1999 eTable 10. Distribution of the Observed Weighted Sample of Early-Neonatal, Late-Neonatal, Postneonatal, and Child Deaths Across All States/Union Territories, 2006 eTable 11. Distribution of the Observed Weighted Sample of Early-Neonatal, Late-Neonatal, Postneonatal, and Child Deaths Across All States/Union Territories, 2016 eTable 12. Distribution of the Observed Weighted Sample of Early-Neonatal, Late-Neonatal, Postneonatal, and Child Deaths Across All States/Union Territories, 2021 eFigure 1. Relationship Between Standardized Absolute Change (1993-2021) and Baseline Early-Neonatal, Late-Neonatal, Postneonatal and Child Mortality Rates (1993) Across States/Union Territories [file jamanetwopen-e2410046-s001.pdf]

Supplementary Online Content

Subramanian SV, Kumar A, Pullum TW, Ambade M, Rajpal S, Kim R. Neonatal and child mortality rates across India, 1993-2021. *JAMA Netw Open*. 2024;7(5):e2410046. doi:10.1001/jamanetworkopen.2024.10046

eTable 1. Early-Neonatal Mortality Rate and 95% Confidence Intervals for States/Union Territories of India, 1993-2021
eTable 2. Late-Neonatal Mortality Rate and 95% Confidence Intervals for States/Union Territories of India, 1993-2021
eTable 3. Postneonatal Mortality Rate and 95% Confidence Intervals for States/Union Territories of India, 1993-2021
eTable 4. Child Mortality Rate and 95% Confidence Intervals for States/Union Territories of India, 1993-2021
eTable 5. Pearson Correlation Coefficient between Early-Neonatal, Late-Neonatal, Postneonatal and Child Mortality Rates Across States/Union Territories, 1993 and 2021
eTable 6. Standard Deviation (SD) and Interquartile Range (IQR) of Early-Neonatal, Late-Neonatal, Postneonatal and Child Mortality Rates of States/Union Territories of India, 1993-2021
eTable 7. Percentage Share of the Burden of Early-Neonatal, Late-Neonatal, Postneonatal and Child Mortality to Under 5 Mortality Across States/Union Territories of India, 1993-2021
eTable 8. Distribution of the Observed Weighted Sample of Early-Neonatal, Late-Neonatal, Postneonatal, and Child Deaths Across All States/Union Territories, 1993
eTable 9. Distribution of the Observed Weighted Sample of Early-Neonatal, Late-Neonatal, Postneonatal, and Child Deaths Across All States/Union Territories, 1999
eTable 10. Distribution of the Observed Weighted Sample of Early-Neonatal, Late-Neonatal, Postneonatal, and Child Deaths Across All States/Union Territories, 2006
eTable 11. Distribution of the Observed Weighted Sample of Early-Neonatal, Late-Neonatal, Postneonatal, and Child Deaths Across All States/Union Territories, 2016
eTable 12. Distribution of the Observed Weighted Sample of Early-Neonatal, Late-Neonatal, Postneonatal, and Child Deaths Across All States/Union Territories, 2021
eFigure 1. Relationship Between Standardized Absolute Change (1993-2021) and Baseline Early-Neonatal, Late-Neonatal, Postneonatal and Child Mortality Rates (1993) Across States/Union Territories of India
eFigure 2. Interactive Dashboard Showing Geographic Distribution of Early-Neonatal, Late-Neonatal, Postneonatal and Child Mortality Rates Across the States and Union Territories of India, 1993-2021
eMethods
eMethods 1. Systematic Search of Prior Literature
eReferences
eMethods 2. National Policies That May Directly or Indirectly Influence Childhood Mortality in India
eMethods 3. Stata Codes to Estimate Early-Neonatal, Late-Neonatal, Postneonatal and Child Mortality Rates Across States/Union Territories of India, 1993 to 2021

This supplementary material has been provided by the authors to give readers additional information about their work.

© 2024 Subramanian SV et al. *JAMA Network Open.*

eTable 1: Early-neonatal Mortality Rate and 95% Confidence Intervals for States/Union Territories of India, 1993-2021

States/Union Territories	1993	1999	2006	2016	2021
**India**	33.5 [32.9-34.1]	31.4 [30.8-32]	29.6 [28.9-30.3]	24.2 [23.4-25]	20.3 [19.6-21.1]
Andhra Pradesh	39 [35.7-42.5]	36.9 [33.2-40.9]	31.9 [28.8-35.3]	20.7 [16.4-26.1]	14.8 [10.7-20.6]
Arunachal Pradesh	11 [8.8-13.7]	30.5 [26.9-34.7]	23.4 [20.1-27.1]	10.5 [7.2-15.3]	6.5 [4.7-9.1]
Assam	28.5 [26.1-31.1]	30.5 [27.9-33.3]	30.6 [27.7-33.8]	26.2 [22.8-30.1]	18.7 [15.5-22.7]
Bihar	41.4 [39.1-43.9]	37.8 [35.8-39.8]	31.7 [29.1-34.5]	31.3 [28.8-33.9]	28.9 [26.3-31.8]
Chhattisgarh	33.8 [28.9-39.5]	37.3 [32.4-42.9]	41.8 [38.5-45.5]	34.2 [29.9-39.1]	26.2 [21.5-31.9]
Goa	14.9 [12.9-17.2]	22.6 [18.7-27.2]	5.8 [4.4-7.7]	11.1 [4.3-28.3]	2.2 [0.3-16.4]
Gujarat	26.2 [24.1-28.5]	30.3 [27.9-32.8]	27.7 [25-30.7]	22.1 [18.2-26.8]	17.2 [13.9-21.2]
Haryana	25 [22.8-27.4]	22.2 [20-24.7]	19.5 [17.1-22.3]	16.8 [13.6-20.7]	18 [14.8-21.9]
Himachal Pradesh	21.4 [19.1-24]	16.8 [14.6-19.4]	19.3 [16.3-22.8]	21 [16.3-27]	16.6 [11.8-23.4]
Jharkhand	33.5 [27.9-40.2]	26.9 [23.8-30.5]	39.1 [35.9-42.6]	27 [23.9-30.5]	23.9 [20.8-27.4]
Karnataka	36.6 [34.3-39]	25.5 [23.3-27.8]	22.5 [20.4-24.7]	15.3 [12.5-18.6]	13.9 [10.8-17.9]
Kerala	12.8 [11.2-14.6]	12.4 [10.4-14.8]	9.4 [7.5-11.6]	3.6 [1.8-7.5]	2 [0.9-4.6]
Madhya Pradesh	36.7 [34.3-39.3]	35.4 [33.5-37.4]	31.1 [28.7-33.7]	29.5 [27.1-32.2]	23.2 [20.7-26]
Maharashtra	27.7 [25.6-30]	25.1 [22.9-27.4]	26.2 [23.9-28.6]	13.5 [10.6-17.3]	12.7 [10.1-15.8]
Manipur	21.5 [18.2-25.4]	13.8 [11.6-16.3]	16.1 [14.2-18.2]	13.2 [10.4-16.8]	14.1 [9.9-20.2]
Meghalaya	27.9 [24.5-31.7]	29.2 [25.9-32.9]	18.8 [16.1-21.9]	16.7 [12.3-22.7]	17.7 [13.1-23.9]
Mizoram	6.2 [4.4-8.8]	15.6 [12.9-18.8]	12.7 [10.3-15.7]	8.4 [4.7-14.9]	10.1 [5.1-19.6]
Nagaland	5.9 [4.4-8]	13.4 [10.9-16.4]	13.8 [12.1-15.7]	15.4 [11.8-20.2]	7.6 [5.1-11.3]
Odisha	39.5 [37-42.2]	34.1 [31.7-36.6]	30.6 [27.8-33.6]	22.1 [19.2-25.4]	19.8 [16.7-23.5]
Punjab	21.8 [19.7-24.2]	22.6 [20.2-25.4]	20.5 [18-23.3]	17.4 [13.6-22.2]	17.2 [13-22.7]
Rajasthan	21.7 [20.1-23.4]	33.7 [32-35.4]	34.3 [31.6-37.2]	24.6 [21.9-27.6]	16.3 [14-18.9]
Sikkim	-	18.8 [15.8-22.4]	11 [8.5-14.3]	17.2 [9.8-29.8]	4.7 [1.3-17.2]
Tamil Nadu	35.5 [32.9-38.3]	28 [25.7-30.6]	13.4 [11.5-15.6]	11.9 [9.4-15]	11.3 [8.7-14.8]
Telangana	32.6 [28.9-36.8]	30.7 [26.9-35]	31.9 [28.8-35.3]	18.5 [13.8-24.7]	14.3 [11.5-17.6]
Tripura	28.6 [24.7-33.1]	25.1 [21-30.1]	28.9 [24.6-34]	9.3 [5.4-15.9]	19.7 [13.5-28.7]
Uttar Pradesh	38.5 [37-40.1]	39.7 [38.1-41.4]	35.6 [34-37.3]	36.6 [34.5-38.8]	29.3 [27.4-31.4]
Uttarakhand	20.6 [17.2-24.6]	13.1 [10.3-16.7]	23.2 [20.5-26.4]	21 [16.9-26.2]	24.6 [17.5-34.6]
West Bengal	37.5 [35-40.1]	22.1 [19.8-24.6]	26.8 [24.4-29.5]	18.2 [14.5-22.7]	12.9 [9.8-16.9]
Andaman & Nicobar Island (UT)	-	-	-	5.6 [1.4-22.1]	8.9 [1.7-45.3]
Chandigarh (UT)	-	-	-	19.9 [7.9-49.1]	1.5 [0.2-11.5]
Dadra Nagar Haveli and Daman & Diu (UT)	-	-	-	8.2 [3.7-18]	15.9 [8.3-30.5]
Jammu & Kashmir (UT)	21.5 [19.3-24]	30.8 [28.2-33.7]	17.7 [15.3-20.4]	18.8 [15.2-23.2]	7.3 [5-10.7]
Ladakh (UT)	-	-	17.7 [15.3-20.4]	24 [13.2-43.5]	11.1 [3.9-31]
Lakshadweep (UT)	-	-	-	19.5 [7.6-49.2]	0 [0-0]
NCT Delhi (UT)	25 [23-27.2]	25.7 [23-28.7]	21.1 [18.4-24.1]	11.5 [6.7-19.6]	12.8 [9-18.2]
Puducherry (UT)	-	-	-	4.8 [2-11.6]	2.2 [0.9-5.6]

© 2024 Subramanian SV et al. *JAMA Network Open.*

eTable 2: Late-neonatal Mortality Rate and 95% Confidence Intervals for States/Union Territories of India, 1993-2021

States/Union Territories	1993	1999	2006	2016	2021
**India**	14.1 [13.8-14.5]	10.7 [10.4-11.1]	8.5 [8.1-8.8]	4.8 [4.4-5.1]	4.1 [3.7-4.4]
Andhra Pradesh	8.7 [7.2-10.5]	10 [8.2-12.3]	7 [5.6-8.8]	2.5 [1.3-4.8]	4.6 [2.6-7.9]
Arunachal Pradesh	6.1 [4.5-8.3]	10.6 [8.4-13.3]	8.6 [6.8-11]	1.2 [0.5-3.2]	1 [0.4-2.5]
Assam	20.6 [18.6-23]	11.2 [9.7-13]	14.4 [12.4-16.8]	6 [4.2-8.5]	3.3 [2.3-4.9]
Bihar	12.4 [11.2-13.9]	9.4 [8.4-10.5]	6.7 [5.6-8.1]	4.9 [4-5.9]	4.9 [3.9-6.1]
Chhattisgarh	7 [5.2-9.5]	16.2 [13.1-20.1]	7.6 [6.3-9.3]	6.7 [4.9-9]	5.9 [4-8.7]
Goa	5.4 [4.3-6.9]	8.5 [6.3-11.6]	2.8 [1.9-4.2]	1.7 [0.2-12.3]	3.3 [0.4-24.3]
Gujarat	15.6 [14-17.4]	8.7 [7.4-10.1]	5.6 [4.4-7]	4.2 [2.6-6.8]	4.3 [3.1-6.1]
Haryana	13.3 [11.7-15.1]	11.1 [9.6-12.9]	3.8 [2.8-5.1]	5 [3.5-7.1]	3.1 [1.9-4.9]
Himachal Pradesh	11.8 [10.1-13.8]	4.9 [3.7-6.4]	7.4 [5.7-9.8]	4.3 [2.2-8.3]	3.4 [1.5-7.6]
Jharkhand	16.8 [12.9-21.7]	9.7 [7.8-12]	9.1 [7.6-10.9]	5.3 [4-6.9]	3.7 [2.5-5.4]
Karnataka	7.9 [6.8-9.1]	10.3 [8.9-11.8]	5.2 [4.3-6.4]	2.7 [1.4-5.1]	1.6 [0.9-2.9]
Kerala	2.4 [1.7-3.2]	1.5 [0.9-2.5]	1.9 [1.2-3.1]	0.7 [0.2-2.8]	1.4 [0.5-3.8]
Madhya Pradesh	18.3 [16.6-20.2]	18.1 [16.8-19.5]	13 [11.4-14.7]	6.7 [5.6-8.2]	4.8 [3.8-6.1]
Maharashtra	8 [6.9-9.3]	6.7 [5.6-7.9]	4.9 [3.9-6]	2.5 [1.3-5.1]	3.5 [2-6]
Manipur	2.7 [1.7-4.4]	4.3 [3.2-5.8]	2.3 [1.7-3.2]	2.1 [1.1-4.1]	3.2 [1.6-6.2]
Meghalaya	8.6 [6.7-10.9]	21 [18.2-24.1]	4.7 [3.4-6.5]	1 [0.5-2.3]	1.5 [0.6-3.8]
Mizoram	1.6 [0.8-3.1]	2.8 [1.7-4.5]	3.3 [2.2-5]	2.7 [0.8-8.3]	1.2 [0.4-3.4]
Nagaland	3.6 [2.4-5.2]	6.1 [4.5-8.3]	5.5 [4.4-6.8]	0.9 [0.3-2.3]	2.4 [1.1-5.2]
Odisha	23.1 [21.2-25.2]	13.4 [12-15]	13.9 [12.1-16]	5.8 [4.2-7.8]	6.6 [4.5-9.6]
Punjab	8.8 [7.5-10.4]	11.2 [9.5-13.2]	6.6 [5.3-8.3]	3.4 [2.1-5.8]	4 [2.6-6.3]
Rajasthan	14.7 [13.3-16.1]	14.5 [13.4-15.7]	9.2 [7.8-10.8]	4.9 [3.9-6.2]	3.4 [2.6-4.6]
Sikkim	-	6.3 [4.6-8.5]	7.8 [5.7-10.7]	3.1 [1-10]	0.2 [0-1.4]
Tamil Nadu	9.5 [8.2-11.1]	6 [4.9-7.3]	5 [3.7-6.6]	1.8 [1-3.2]	1.2 [0.5-2.6]
Telangana	7.4 [5.7-9.5]	5.8 [4.3-7.8]	7 [5.6-8.8]	1.3 [0.4-3.7]	2 [1.3-3.2]
Tripura	13.6 [11-16.8]	3.4 [2.1-5.5]	2.7 [1.6-4.5]	3.3 [1.1-10]	2.5 [1-6.5]
Uttar Pradesh	21.1 [20-22.3]	13.3 [12.4-14.2]	11 [10.1-12]	7.9 [6.9-8.9]	5.7 [4.8-6.6]
Uttarakhand	20.4 [16.6-25.1]	12 [9-15.8]	3.3 [2.3-4.6]	7.1 [5-10.1]	7.1 [4.3-11.7]
West Bengal	13.5 [12-15.1]	9.5 [8-11.2]	9.3 [7.9-10.9]	3.4 [2-5.8]	2.5 [1.5-4.2]
Andaman & Nicobar Island (UT)	-	-	-	1.5 [0.2-10.6]	3.1 [0.4-23]
Chandigarh (UT)	-	-	-	9.9 [2.4-39.8]	0 [0-0]
Dadra Nagar Haveli and Daman & Diu (UT)	-	-	-	5.5 [2-15.1]	5 [1.7-14.9]
Jammu & Kashmir (UT)	9.5 [8.1-11.3]	8.9 [7.5-10.5]	11.7 [9.8-13.9]	3.9 [2.1-7.2]	2.4 [1-5.7]
Ladakh (UT)	-	-	11.7 [9.8-13.9]	1.3 [0.2-9.3]	0 [0-0]
Lakshadweep (UT)	-	-	-	3.2 [0.4-23.3]	0 [0-0]
NCT Delhi (UT)	9.4 [8.2-10.9]	3.6 [2.6-4.9]	7.4 [5.9-9.2]	6.1 [2.6-14.3]	4.2 [2.2-8.3]
Puducherry (UT)	-	-	-	0.9 [0.2-4.7]	0 [0-0]

© 2024 Subramanian SV et al. *JAMA Network Open.*

eTable 3: Postneonatal Mortality Rate and 95% Confidence Intervals for States/Union Territories of India, 1993-2021

States/Union Territories	1993	1999	2006	2016	2021
**India**	31 [31-31]	25.3 [25.3-25.3]	18.9 [18.9-18.9]	11.7 [11.7-11.7]	10.8 [10.8-10.8]
Andhra Pradesh	30.9 [30.9-30.9]	30.9 [30.9-31]	14.6 [14.6-14.6]	11.7 [11.7-11.7]	10.8 [10.8-10.8]
Arunachal Pradesh	22.9 [22.9-22.9]	22 [22-22]	28.7 [28.7-28.7]	11.2 [11.2-11.2]	5.3 [5.3-5.3]
Assam	39.6 [39.6-39.6]	27.8 [27.8-27.8]	21.1 [21.1-21.1]	15.4 [15.4-15.4]	9.9 [9.9-9.9]
Bihar	37.3 [37.3-37.3]	30.7 [30.7-30.7]	23.3 [23.3-23.3]	12 [12-12]	13 [13-13]
Chhattisgarh	23.6 [23.6-23.7]	27.3 [27.3-27.3]	21.3 [21.3-21.3]	13.1 [13.1-13.1]	12.1 [12.1-12.2]
Goa	11.6 [11.6-11.6]	5.6 [5.6-5.6]	6.7 [6.7-6.7]	0.2 [0.2-0.2]	0.1 [0.1-0.1]
Gujarat	26.9 [26.9-26.9]	23.7 [23.7-23.7]	16.4 [16.4-16.4]	7.9 [7.9-7.9]	9.7 [9.7-9.7]
Haryana	35 [35-35]	23.4 [23.4-23.5]	18.4 [18.4-18.4]	11.1 [11-11.1]	12.2 [12.2-12.2]
Himachal Pradesh	22.6 [22.6-22.6]	12.7 [12.7-12.7]	9.4 [9.4-9.4]	9 [9-9]	5.6 [5.6-5.6]
Jharkhand	25 [25-25]	17.6 [17.6-17.7]	20.5 [20.5-20.5]	11.4 [11.4-11.4]	10.3 [10.3-10.3]
Karnataka	21 [21-21]	15.7 [15.7-15.7]	15.5 [15.5-15.5]	8.9 [8.9-8.9]	9.9 [9.8-9.9]
Kerala	8.6 [8.6-8.6]	2.4 [2.4-2.4]	4.1 [4.1-4.1]	1.3 [1.3-1.3]	1.1 [1.1-1.1]
Madhya Pradesh	36.6 [36.6-36.6]	34.1 [34.1-34.1]	25.4 [25.4-25.4]	14.9 [14.9-14.9]	13.3 [13.3-13.3]
Maharashtra	14.8 [14.8-14.8]	12 [12-12]	6.5 [6.5-6.5]	7.6 [7.6-7.6]	7.1 [7.1-7.1]
Manipur	18.2 [18.2-18.2]	18.9 [18.9-18.9]	11.3 [11.3-11.3]	6.4 [6.4-6.4]	7.6 [7.6-7.7]
Meghalaya	27.7 [27.7-27.7]	38.8 [38.8-38.8]	21 [21-21]	12.1 [12.1-12.1]	13.1 [13.1-13.1]
Mizoram	6.8 [6.8-6.8]	18.7 [18.7-18.7]	18 [18-18]	29 [29-29]	10.1 [10.1-10.1]
Nagaland	7.7 [7.7-7.7]	22.6 [22.6-22.6]	19 [19-19]	13.2 [13.2-13.2]	13.5 [13.5-13.5]
Odisha	49.5 [49.5-49.5]	33.4 [33.4-33.4]	20.2 [20.2-20.2]	11.8 [11.7-11.8]	9.9 [9.9-9.9]
Punjab	23.1 [23.1-23.1]	23.3 [23.3-23.3]	14.5 [14.5-14.6]	8.4 [8.4-8.4]	6.8 [6.8-6.8]
Rajasthan	36.2 [36.2-36.3]	32.1 [32.1-32.1]	21.8 [21.8-21.8]	11.8 [11.8-11.8]	10.5 [10.5-10.5]
Sikkim	-	18.8 [18.8-18.8]	14.9 [14.9-14.9]	9.2 [9.2-9.2]	6.3 [6.3-6.3]
Tamil Nadu	23.1 [23.1-23.2]	14.2 [14.2-14.2]	12 [12-12]	6.5 [6.5-6.5]	6.1 [6.1-6.1]
Telangana	17.4 [17.4-17.4]	13.7 [13.7-13.7]	14.6 [14.6-14.6]	7.9 [7.9-7.9]	10.1 [10.1-10.1]
Tripura	33.6 [33.6-33.6]	15.7 [15.7-15.7]	19.8 [19.8-19.8]	14 [14-14]	15.3 [15.3-15.3]
Uttar Pradesh	42.3 [42.2-42.3]	36 [36-36]	26 [26-26]	19 [19-19]	15.3 [15.3-15.3]
Uttarakhand	23.6 [23.6-23.6]	12.7 [12.7-12.7]	15.4 [15.4-15.4]	11.6 [11.6-11.6]	7.4 [7.4-7.4]
West Bengal	24.3 [24.3-24.3]	17.2 [17.1-17.2]	11.9 [11.9-11.9]	6 [6-6]	6.6 [6.6-6.6]
Andaman & Nicobar Island (UT)	-	-	-	2.8 [2.8-2.8]	8.6 [8.6-8.6]
Chandigarh (UT)	-	-	-	8.5 [8.5-8.5]	14 [14-14]
Dadra Nagar Haveli and Daman & Diu (UT)	-	-	-	19.6 [19.6-19.6]	10.9 [10.9-10.9]
Jammu & Kashmir (UT)	14.4 [14.4-14.4]	25.2 [25.2-25.2]	15.3 [15.3-15.3]	9.7 [9.7-9.7]	6.5 [6.5-6.5]
Ladakh (UT)	-	-	15.3 [15.3-15.3]	9.9 [9.9-9.9]	8.9 [8.9-9]
Lakshadweep (UT)	-	-	-	4.3 [4.2-4.3]	0 [0-0]
NCT Delhi (UT)	30.9 [30.9-30.9]	17.6 [17.6-17.6]	11.3 [11.3-11.3]	13.7 [13.7-13.7]	7.4 [7.4-7.4]
Puducherry (UT)	-	-	-	10 [10-10]	0.7 [0.7-0.7]

© 2024 Subramanian SV et al. *JAMA Network Open.*

eTable 4: Child Mortality Rate and 95% Confidence Intervals for States/Union Territories of India, 1993-2021

States/Union Territories	1993	1999	2006	2016	2021
**India**	33.5 [31.7-35.2]	29.2 [27.6-30.8]	18.4 [16.9-19.8]	9.4 [8.9-9.9]	6.9 [6.4-7.4]
Andhra Pradesh	24.8 [17.1-32.5]	25 [16.1-34]	10.2 [5.3-15.2]	6.1 [3.1-9]	5.1 [2.4-7.8]
Arunachal Pradesh	33.3 [20.6-45.9]	37.3 [25.3-49.3]	28.7 [18.4-39.1]	10.2 [7.4-13]	6 [3.9-8.2]
Assam	58.6 [48.3-69]	21.4 [15.1-27.7]	20.2 [13.3-27.2]	9.3 [7.3-11.3]	7.4 [5.5-9.4]
Bihar	43.5 [36.5-50.4]	37.3 [31.8-42.9]	24.6 [18.1-31.2]	10.5 [9.1-11.8]	10.2 [8.6-11.7]
Chhattisgarh	26.2 [13.1-39.3]	45.4 [29.4-61.5]	21 [14-28]	10.9 [8.4-13.3]	6.4 [4.2-8.7]
Goa	7.2 [3.1-11.3]	10.5 [2-19]	5 [0.8-9.2]	0 [0-0]	5 [0-11.8]
Gujarat	37.8 [30.1-45.5]	24 [17.6-30.4]	11.9 [6.7-17.1]	9.6 [7-12.2]	6.6 [4.9-8.3]
Haryana	27.4 [20.4-34.4]	21.2 [14.7-27.6]	11.1 [5.5-16.7]	8.6 [6.2-11]	5.6 [3.8-7.3]
Himachal Pradesh	14.1 [8.3-19.9]	8.3 [3.5-13.2]	5.6 [0.6-10.6]	3.5 [1.3-5.6]	3.4 [0.9-5.9]
Jharkhand	33.5 [16.6-50.5]	25.4 [16.5-34.2]	26.1 [18.4-33.7]	11.1 [8.9-13.2]	7.9 [6-9.7]
Karnataka	23.6 [18.2-29]	19.3 [13.8-24.9]	12.1 [7.8-16.4]	4.7 [2.8-6.7]	4.2 [2.8-5.7]
Kerala	8.4 [4.6-12.1]	2.6 [0-5.2]	1 [0-2.9]	1.5 [0-3.3]	0.8 [0-1.6]
Madhya Pradesh	56.6 [47.8-65.5]	59.7 [52.8-66.7]	26.5 [20.2-32.9]	14.2 [12.5-15.8]	8.2 [6.7-9.8]
Maharashtra	21.3 [15.8-26.8]	15 [10-20]	9.5 [5.4-13.6]	5.2 [2.7-7.6]	4.9 [2.5-7.3]
Manipur	20.2 [10.6-29.7]	19.9 [11.7-28]	12.5 [7.5-17.6]	4.3 [2.6-5.9]	5.2 [2.3-8.1]
Meghalaya	24.3 [14.5-34]	36.2 [24.2-48.2]	27.1 [16.9-37.3]	10.1 [6.8-13.4]	8 [5.3-10.6]
Mizoram	14.9 [6-23.9]	18.3 [9.5-27.2]	19.5 [10.3-28.6]	6.1 [3.2-9]	2.8 [1-4.6]
Nagaland	3.6 [0-7.6]	22.6 [12.2-33.1]	27.5 [19.7-35.3]	8.2 [5.1-11.2]	9.8 [6-13.6]
Odisha	21.3 [15.9-26.6]	25.5 [19.5-31.6]	27.6 [19.7-35.5]	8.8 [6.8-10.8]	4.9 [3.3-6.6]
Punjab	15 [9.7-20.4]	15.9 [9.8-22.1]	10.8 [5.3-16.3]	4.1 [2.5-5.7]	4.8 [2.9-6.8]
Rajasthan	32.3 [26.7-37.9]	37.6 [32.5-42.6]	21.5 [15.2-27.8]	9.7 [8.1-11.4]	7.5 [5.4-9.7]
Sikkim	-	28.4 [16.5-40.2]	6.7 [0.5-12.8]	2.8 [0-5.7]	0 [0-0]
Tamil Nadu	20.1 [14.1-26.1]	15.9 [10.3-21.5]	5.3 [1.7-9]	6.7 [4.2-9.2]	3.7 [2.2-5.2]
Telangana	18.7 [10.3-27.2]	15.9 [8.1-23.7]	10.2 [5.3-15.2]	4.2 [1.3-7]	3 [1.2-4.9]
Tripura	31.2 [19.3-43]	7.4 [1.2-13.5]	8.2 [1-15.4]	6.1 [1.7-10.6]	5.9 [2.5-9.4]
Uttar Pradesh	47 [41.9-52]	40.2 [35.6-44.8]	25.6 [21.7-29.5]	15.6 [14.1-17]	10 [8.8-11.2]
Uttarakhand	30.4 [16.4-44.4]	19.3 [7.8-30.9]	15.5 [8.9-22.1]	7.1 [4.7-9.4]	6.7 [3.1-10.3]
West Bengal	26 [20.2-31.9]	19.9 [13.6-26.3]	12.2 [7.2-17.1]	4.4 [2.5-6.3]	3.4 [1.8-5.1]
Andaman & Nicobar Island (UT)	-	-	-	3.2 [0-7.6]	4 [0-10.6]
Chandigarh (UT)	-	-	-	0 [0-0]	4.2 [0-12.7]
Dadra Nagar Haveli and Daman & Diu (UT)	-	-	-	6.8 [0-14]	5.3 [0.2-10.5]
Jammu & Kashmir (UT)	14.3 [8.6-20]	16.1 [10.5-21.7]	6.8 [2.6-11.1]	5.4 [3.2-7.5]	2.3 [1.1-3.5]
Ladakh (UT)	-	-	6.8 [2.6-11.1]	5.5 [0.3-10.7]	9.7 [1-18.4]
Lakshadweep (UT)	-	-	-	3.3 [0-9.8]	0 [0-0]
NCT Delhi (UT)	19 [13.5-24.4]	9 [4.3-13.6]	7.3 [3.1-11.4]	11.3 [4.9-17.8]	6.3 [3.4-9.3]
Puducherry (UT)	-	-	-	0.5 [0-1]	1 [0-2.9]

© 2024 Subramanian SV et al. *JAMA Network Open.*

eTable 5: Pearson Correlation Coefficient between Early-neonatal, Late-neonatal, Postneonatal and Child Mortality Rates across States/Union Territories, 1993 and 2021

1993 (Observations: 29)	**Early-neonatal**	**Late-neonatal**	**Postneonatal**	**Child**
**Early-neonatal**	1.00	-	-	-
**Late-neonatal**	0.55	1.00	-	-
**Postneonatal**	0.66	0.81	1.00	-
**Child**	0.52	0.70	0.71	1.00

2021 (Observations: 36)	**Early-neonatal**	**Late-neonatal**	**Postneonatal**	**Child**
**Early-neonatal**	1.00	-	-	-
**Late-neonatal**	0.77	1.00	-	-
**Postneonatal**	0.65	0.35	1.00	-
**Child**	0.67	0.47	0.67	1.00

Note: All values are significant to $p<0.01$, and values are rounded to 2 decimal places.

© 2024 Subramanian SV et al. *JAMA Network Open.*

eTable 6: Standard Deviation (SD) and Interquartile Range (IQR) of Early-neonatal, Late-neonatal, Postneonatal and Child Mortality Rates of States/Union Territories of India, 1993-2021

Survey Year	Early-neonatal		Late-neonatal		Postneonatal		Child	
	SD	IQR	SD	IQR	SD	IQR	SD	IQR
1993	9.9	13.6	5.9	7	10.6	14.1	13.2	14.2
1999	7.9	9	4.5	5.2	8.8	11.9	12.4	12.9
2006	9	13	3.4	4.3	5.8	6.7	8.4	14.3
2016	8	10.2	2.3	3.5	5.2	4.2	3.8	5.5
2021	7.7	9.8	1.9	2.8	4	4.4	2.7	3.2

Note: Values are expressed in per 1,000 live births and are rounded to 1 decimal place.

© 2024 Subramanian SV et al. *JAMA Network Open.*

eTable 7: Percentage Share of the Burden of Early-neonatal, Late-neonatal, Postneonatal and Child Mortality to Under 5 Mortality across States/Union Territories of India, 1993-2021

	Early-neonatal		Late-neonatal		Post-neonatal		Child	
	1993	2021	1993	2021	1993	2021	1993	2021
Andhra Pradesh	37.7	42.0	8.4	13.0	29.9	30.6	24.0	14.4
Arunachal Pradesh	15.0	34.6	8.3	5.3	31.3	28.2	45.4	31.9
Assam	19.3	47.6	14.0	8.5	26.9	25.0	39.8	18.9
Bihar	30.8	50.8	9.2	8.6	27.7	22.8	32.3	17.8
Chhattisgarh	37.2	51.7	7.7	11.6	26.1	24.0	28.9	12.7
Goa	38.1	20.9	13.9	30.8	29.6	1.3	18.4	47.0
Gujarat	24.6	45.4	14.6	11.5	25.2	25.6	35.5	17.5
Haryana	24.8	46.4	13.2	7.9	34.8	31.5	27.2	14.3
Himachal Pradesh	30.6	57.3	16.8	11.7	32.4	19.2	20.1	11.7
Jharkhand	30.8	52.2	15.4	8.1	23.0	22.5	30.8	17.2
Karnataka	41.1	46.9	8.8	5.5	23.6	33.3	26.5	14.2
Kerala	39.7	38.1	7.4	26.5	26.9	20.5	26.0	14.9
Madhya Pradesh	24.7	46.8	12.4	9.7	24.7	26.9	38.2	16.7
Maharashtra	38.6	45.0	11.1	12.3	20.6	25.2	29.7	17.5
Manipur	34.4	46.9	4.3	10.6	29.1	25.4	32.2	17.2
Meghalaya	31.5	44.0	9.7	3.7	31.3	32.5	27.5	19.8
Mizoram	21.1	41.7	5.3	4.9	23.0	41.8	50.5	11.6
Nagaland	28.6	22.8	17.1	7.1	37.2	40.6	17.2	29.5
Odisha	29.6	48.1	17.3	16.0	37.1	23.9	15.9	12.0
Punjab	31.7	52.4	12.8	12.3	33.6	20.6	21.9	14.7
Rajasthan	20.7	43.2	14.0	9.0	34.6	27.9	30.8	19.9
Sikkim		42.2		1.7		56.1		0.0
Tamil Nadu	40.2	50.7	10.8	5.3	26.2	27.4	22.8	16.7
Telangana	42.9	48.4	9.7	7.0	22.9	34.3	24.6	10.3
Tripura	26.7	45.4	12.7	5.8	31.4	35.1	29.1	13.7
Uttar Pradesh	25.9	48.6	14.2	9.4	28.4	25.4	31.5	16.5
Uttarakhand	21.7	53.8	21.5	15.5	24.9	16.1	32.0	14.6
West Bengal	37.0	50.8	13.3	9.9	24.0	25.8	25.7	13.5
Andaman & Nicobar Island (UT)		36.4		12.7		34.8		16.2
Chandigarh (UT)		7.7		0.0		70.8		21.5
Dadra Nagar Haveli and Daman & Diu (UT)		42.9		13.4		29.4		14.3
Jammu & Kashmir (UT)	36.0	39.3	16.0	13.1	24.0	35.2	24.0	12.4
Ladakh (UT)		37.2		0.0		30.1		32.7
Lakshadweep (UT)		0.0		0.0		0.0		0.0
NCT Delhi (UT)	29.7	41.6	11.2	13.8	36.6	24.1	22.5	20.5
Puducherry (UT)		56.5		0.0		18.4		25.1

© 2024 Subramanian SV et al. *JAMA Network Open.*

eTable 8: Distribution of the Observed Weighted Sample of Early-neonatal, Late-neonatal, Postneonatal, and Child Deaths across all States/Union Territories, 1993

State/Union Territories	Early-neonatal	Late-neonatal	Postneonatal	Child
Andhra Pradesh	97	22	72	61
Arunachal Pradesh	1	1	2	3
Assam	60	43	78	120
Bihar	260	79	227	258
Chhattisgarh	43	9	28	31
Goa	1	0	1	0
Gujarat	73	44	71	98
Haryana	37	20	49	36
Himachal Pradesh	8	4	9	5
Jammu & Kashmir	6	3	4	4
Jharkhand	30	15	21	31
Karnataka	116	24	64	72
Kerala	19	4	12	12
Madhya Pradesh	149	72	142	205
Maharashtra	150	43	73	110
Manipur	3	0	3	3
Meghalaya	4	1	4	4
Mizoram	0	0	0	1
NCT Delhi	18	7	23	13
Nagaland	1	0	1	0
Odisha	91	54	113	47
Punjab	30	12	30	21
Rajasthan	68	47	115	106
Tamil Nadu	117	31	71	60
Telangana	48	12	29	30
Tripura	6	2	7	7
Uttar Pradesh	494	261	522	518
Uttarakhand	10	10	11	15
West Bengal	174	61	113	118

© 2024 Subramanian SV et al. *JAMA Network Open.*

eTable 9: Distribution of the Observed Weighted Sample of Early-neonatal, Late-neonatal, Postneonatal, and Child Deaths across all States/Union Territories, 1999

States/Union Territories	Early-neonatal	Late-neonatal	Postneonatal	Child
Andhra Pradesh	83	22	65	56
Arunachal Pradesh	2	1	1	3
Assam	44	16	38	32
Bihar	201	51	156	201
Chhattisgarh	44	19	30	52
Goa	1	0	0	0
Gujarat	75	22	58	57
Haryana	25	12	25	23
Himachal Pradesh	5	1	3	2
Jammu & Kashmir	16	4	13	8
Jharkhand	38	14	25	39
Karnataka	65	26	37	49
Kerala	17	2	3	3
Madhya Pradesh	138	71	129	227
Maharashtra	125	34	56	71
Manipur	2	1	2	2
Meghalaya	5	4	7	5
Mizoram	1	0	1	1
NCT Delhi	17	2	11	6
Nagaland	2	1	2	2
Odisha	68	27	63	48
Punjab	24	12	25	18
Rajasthan	126	53	116	135
Sikkim	0	0	1	1
Tamil Nadu	84	18	41	44
Telangana	52	10	22	28
Tripura	4	1	2	1
Uttar Pradesh	452	153	398	442
Uttarakhand	7	6	6	10
West Bengal	87	38	67	82

© 2024 Subramanian SV et al. *JAMA Network Open.*

eTable 10: Distribution of the Observed Weighted Sample of Early-neonatal, Late-neonatal, Postneonatal, and Child Deaths across all States/Union Territories, 2006

States/Union Territories	Early-neonatal	Late-neonatal	Postneonatal	Child
Andhra Pradesh	103	23	44	35
Arunachal Pradesh	2	1	2	2
Assam	45	22	30	30
Bihar	203	43	142	153
Chhattisgarh	52	10	25	26
Delhi	12	4	6	4
Gujarat	75	13	43	31
Goa	0	0	0	0
Himachal Pradesh	5	2	2	1
Haryana	21	4	19	11
Jharkhand	73	18	40	49
Jammu And Kashmir	8	6	7	3
Karnataka	63	15	40	33
Kerala	10	2	4	1
Meghalaya	4	1	4	5
Maharashtra	117	22	27	42
Manipur	2	0	1	1
Madhya Pradesh	123	51	94	101
Mizoram	1	0	1	1
Nagaland	1	1	2	3
Orissa	61	28	39	53
Punjab	24	8	15	12
Rajasthan	122	32	77	75
Sikkim	0	0	0	0
Tamil Nadu	27	11	26	12
Tripura	5	0	3	1
Uttaranchal	10	1	6	7
Uttar Pradesh	426	134	303	302
West Bengal	115	40	46	49

© 2024 Subramanian SV et al. *JAMA Network Open.*

eTable 11: Distribution of the Observed Weighted Sample of Early-neonatal, Late-neonatal, Postneonatal, and Child Deaths across all States/Union Territories, 2016

States/Union Territories	Early-neonatal	Late-neonatal	Postneonatal	Child
Andaman And Nicobar Islands	0	0	0	0
Andhra Pradesh	190	23	102	51
Arunachal Pradesh	2	0	2	2
Assam	165	36	96	58
Bihar	1011	158	367	322
Chandigarh	3	1	1	0
Chhattisgarh	215	42	74	61
Dadra & Nagar Haveli And Daman & Diu	1	1	2	1
Delhi	36	19	42	37
Goa	2	0	0	0
Gujarat	240	47	80	101
Haryana	99	29	61	48
Himachal Pradesh	25	5	10	4
Jammu And Kashmir	42	8	21	12
Jharkhand	207	41	82	82
Karnataka	162	29	90	49
Kerala	16	3	5	7
Ladakh	1	0	0	0
Lakshadweep	0	0	0	0
Madhya Pradesh	518	118	246	233
Maharashtra	293	55	152	105
Manipur	7	1	3	2
Meghalaya	13	1	9	8
Mizoram	2	1	6	1
Nagaland	5	0	4	3
Odisha	184	47	95	69
Puducherry	1	0	2	0
Punjab	73	15	34	17
Rajasthan	367	73	169	141
Sikkim	1	0	1	0
Tamil Nadu	172	27	92	93
Telangana	133	9	57	29
Tripura	6	2	8	4
Uttar Pradesh	1676	362	826	683
Uttarakhand	42	15	24	14
West Bengal	311	56	95	72

© 2024 Subramanian SV et al. *JAMA Network Open.*

eTable 12: Distribution of the Observed Weighted Sample of Early-neonatal, Late-neonatal, Postneonatal, and Child Deaths across all States/Union Territories, 2021

States/Union Territories	Early-neonatal	Late-neonatal	Postneonatal	Child
Andaman & Nicobar Islands	0	0	0	0
Andhra Pradesh	113	38	73	40
Arunachal Pradesh	1	0	1	1
Assam	110	21	56	46
Bihar	912	153	372	305
Chandigarh	0	0	2	1
Chhattisgarh	135	31	60	33
Dadra & Nagar Haveli And Daman & Diu	1	0	1	0
Goa	0	1	0	1
Gujarat	175	44	85	66
Haryana	82	14	51	24
Himachal Pradesh	17	3	4	3
Jammu & Kashmir	12	4	10	4
Jharkhand	171	27	66	56
Karnataka	143	19	99	45
Kerala	9	6	5	4
Ladakh	0	0	0	0
Lakshadweep	0	0	0	0
Madhya Pradesh	330	68	175	114
Maharashtra	244	67	128	99
Manipur	7	1	3	2
Meghalaya	16	1	11	7
Mizoram	2	0	2	1
Nagaland	2	1	3	2
Nct Of Delhi	40	13	20	20
Odisha	150	49	64	37
Puducherry	0	0	0	0
Punjab	72	17	26	20
Rajasthan	251	52	146	113
Sikkim	0	0	0	0
Tamil Nadu	125	13	63	40
Telangana	77	11	49	16
Tripura	12	2	9	4
Uttar Pradesh	1378	268	647	455
Uttarakhand	48	13	10	13
West Bengal	201	41	105	56

© 2024 Subramanian SV et al. *JAMA Network Open.*

eFigure 1: Relationship between Standardized Absolute Change (1993-2021) and baseline Early-neonatal, Late-neonatal, Postneonatal and Child Mortality Rates (1993) across States/Union Territories of India

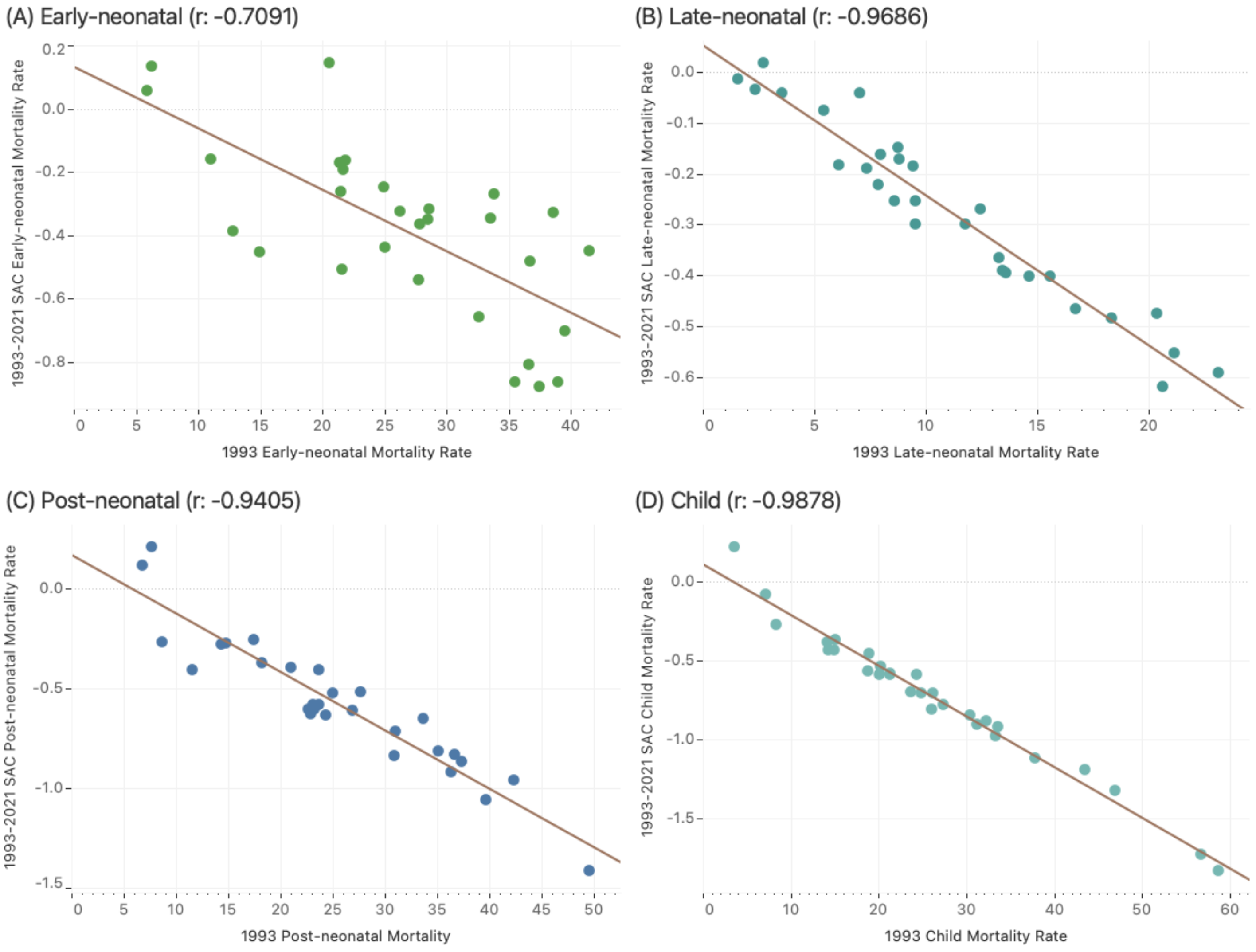

© 2024 Subramanian SV et al. *JAMA Network Open.*

eFigure 2: Interactive Dashboard showing Geographic Distribution of Early-neonatal, Late-neonatal, Postneonatal and Child mortality rates across the States and Union Territories of India, 1993-2021

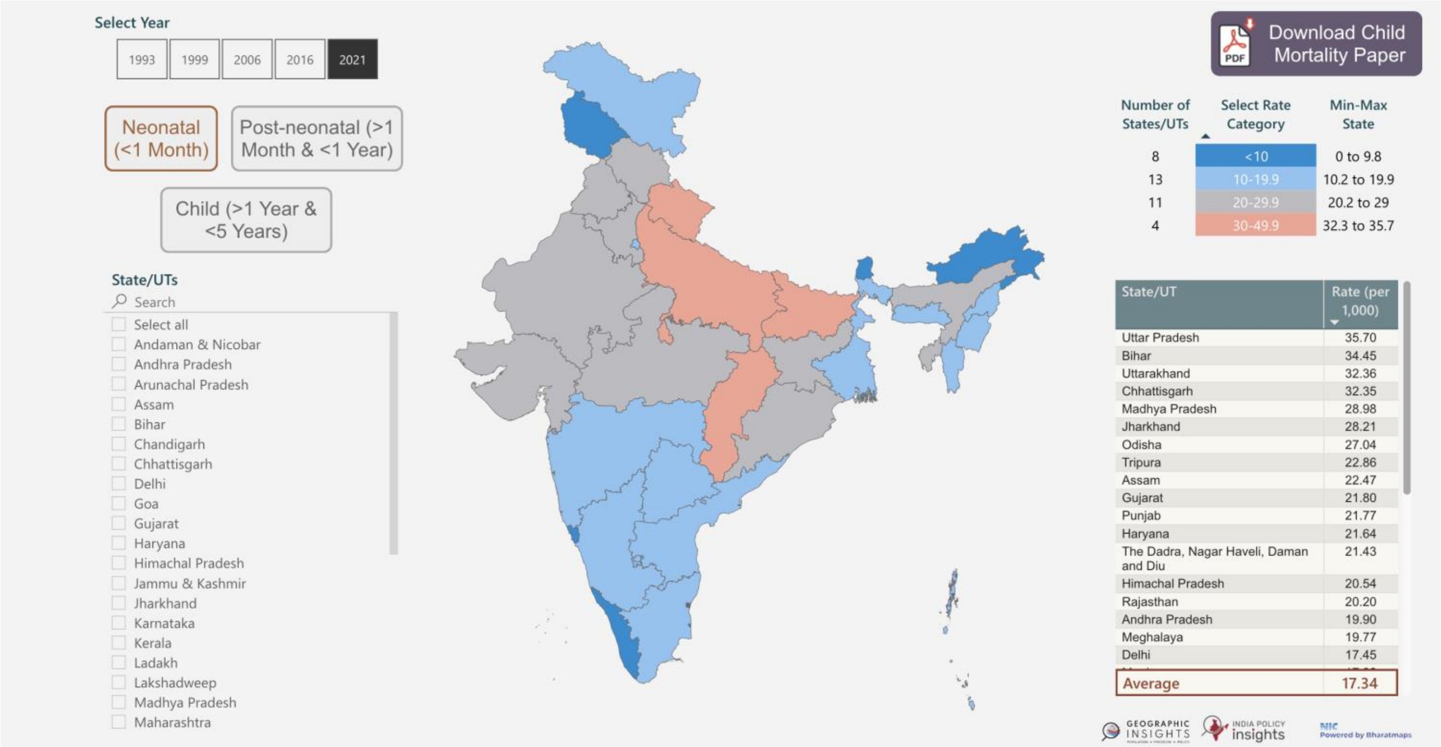

https://geographicinsights.iq.harvard.edu/State-Child-Mortality

© 2024 Subramanian SV et al. *JAMA Network Open.*

eMethods 1: Systematic search of prior literature

We searched PubMed and Web of Sciences to identify empirical studies that conducted quantitative analysis on changes in early life mortality across states/Union Territories (UTs) of India using data for at least two time periods. We used structured combinations of keywords: ("Neonatal Mortality" OR "Postneonatal Mortality" OR "Child Mortality" OR "Infant Mortality" OR "Under-five Mortality" OR "Early Neonatal Mortality" OR "Late Neonatal Mortality") AND "India" AND "States" AND ("Trends" OR "Change"), without any time limitations. The search yielded 2560 studies, we found that 22 studies fit the criteria. Out of the 20 studies meeting our criteria, none provided estimates for Postneonatal mortality, but instead grouped it into broader categories of infant or under-five mortality. Only 7 reported neonatal mortality, and four, child mortality (ages 1-4 years), as defined in our study, and three reported early neonatal mortality. While subnational estimates for PNMR in India are available (Kim et al 2020), notably, we are the first to provide subnational trends for the same. While national estimates for early neonatal mortality were available across two or more time periods, none provided a subnational analysis for the same, except one where only regional estimate were provided in place of state wise estimates. The timelines for all 20 studies ranged from 10-32 years, but mainly used data only up to 2016. A single study by Kumar et al (2022) assessed state-level changes up to 2021, but it only examined under-five mortality. In contrast, we study mortality from a 30-year timeline with data as current as 2021. Further, except for Kumar et al (2022) and Bora and Saikia (2015), none offered estimates across all 36 states and union territories. Importantly, none of these studies considered the changing geography of states and UTs in recent decades. They either looked at unaltered regions or treated bifurcated states as one, rendering comparisons meaningless. In contrast, we utilized DHS geographic data to accurately align values with current state boundaries, thus providing estimates for all 36 states and UTs in past periods based on present state divisions. These distinctive attributes of our study set it apart as the most comprehensive subnational assessment of disaggregated mortality in India.

Details of quantitative subnational (state-level) evidence on early life mortalities in India with data for at least two time periods

Authors	Year	Title	Data source	Citation
**Alderman, H., Nguyen, P. H., & Menon, P.**	2019	Progress in reducing child mortality and stunting in India: an application of the Lives Saved Tool	National Family Health Survey (3&4)	Alderman H, Nguyen PH, Menon P. Progress in reducing child mortality and stunting in India: an application of the Lives Saved Tool. Health Policy Plan. 2019 Nov 1;34(9):667-675. doi: 10.1093/heapol/czz088. PMID: 31529050; PMCID: PMC6880331.
**Alderman, H., Nguyen, P. H., Tran, L. M., & Menon, P.**	2021	Trends and geographic variability in gender inequalities in child mortality and stunting in India, 2006	National Family Health Survey (1-3)	Alderman H, Nguyen PH, Tran LM, Menon P. Trends and geographic variability in gender inequalities in child mortality and stunting in India, 2006-2016. Matern Child Nutr. 2021 Jul;17(3):e13179. doi: 10.1111/mcn.13179. Epub 2021 Mar 14. PMID: 33719159; PMCID: PMC8189201.
**Arokiasamy, P., & Gautam, A.**	2008	Neonatal mortality in the empowered action group states of India: Trends and determinants	Sample Registration Survey, National Family Health Survey	Arokiasamy P, Gautam A. Neonatal mortality in the empowered action group states of India: trends and determinants. J Biosoc Sci. 2008 Mar;40(2):183-201. doi: 10.1017/S0021932007002623. Epub 2007 Dec 20. PMID: 18093346.
**Behl, A. S.**	2013	Trends in child mortality in India	National Family Health Survey (1-3)	Behl AS. Trends in child mortality in India. Indian Pediatr. 2013 Jan 8;50(1):143-7. PMID: 23396786.
**Bhatia, M., Dwivedi, L. K., Ranjan, M., Dixit, P., & Putcha, V.**	2019	Trends, patterns and predictive factors of infant and child mortality in well-performing and underperforming states of India: a secondary analysis using National Family Health Surveys	National Family Health Survey (1-4)	Bhatia M, Dwivedi LK, Ranjan M, Dixit P, Putcha V. Trends, patterns and predictive factors of infant and child mortality in well-performing and underperforming states of India: a secondary analysis using National Family Health Surveys. BMJ Open. 2019 Mar 20;9(3):e023875. doi: 10.1136/bmjopen-2018-023875. PMID: 30898805; PMCID: PMC6475182.

© 2024 Subramanian SV et al. *JAMA Network Open.*

**Bhatia, M., Ranjan, M., Dixit, P., & Dwivedi, L. K.**	2018	Mind the gap: Temporal trends in inequalities in infant and child mortality in India	National Family Health Survey (1-4)	Bhatia M, Ranjan M, Dixit P, Dwivedi LK. Mind the gap: Temporal trends in inequalities in infant and child mortality in India (1992-2016). SSM Popul Health. 2018 May 9;5:201-209. doi: 10.1016/j.ssmph.2018.05.001. PMID: 30094315; PMCID: PMC6072655.
**Bora, J. K., & Saikia, N.**	2018	Neonatal and under-five mortality rate in Indian districts with reference to Sustainable Development Goal 3: An analysis of the National Family Health Survey of India	National Family Health Survey (1-4)	Bora JK, Saikia N. Neonatal and under-five mortality rate in Indian districts with reference to Sustainable Development Goal 3: An analysis of the National Family Health Survey of India (NFHS), 2015-2016. PLoS One. 2018 Jul 30;13(7):e0201125. doi: 10.1371/journal.pone.0201125. PMID: 30059555; PMCID: PMC6066210.
**India State-Level Disease Burden Initiative Child Mortality**	2020	Subnational mapping of under-5 and neonatal mortality trends in India: the Global Burden of Disease Study 2000-17	Multiple data sources	India State-Level Disease Burden Initiative Child Mortality Collaborators. Subnational mapping of under-5 and neonatal mortality trends in India: the Global Burden of Disease Study 2000-17. Lancet. 2020 May 23;395(10237):1640-1658. doi: 10.1016/S0140-6736(20)30471-2. Epub 2020 May 12. PMID: 32413293; PMCID: PMC7262604.
**Kumar, C., Piyasa, & Saikia, N.**	2022	An update on explaining the rural-urban gap in under-five mortality in India	National Family Health Survey (1-5)	Kumar C, Piyasa, Saikia N. An update on explaining the rural-urban gap in under-five mortality in India. BMC Public Health. 2022 Nov 16;22(1):2093. doi: 10.1186/s12889-022-14436-7. PMID: 36384563; PMCID: PMC9670513.
**Makela, S. M., Dandona, R., Dilip, T. R., & Dandona, L.**	2013	Social Sector Expenditure and Child Mortality in India: A State-Level Analysis from 1997 to 2009	Sample Registration Survey, Census	Makela SM, Dandona R, Dilip TR, Dandona L. Social sector expenditure and child mortality in India: a state-level analysis from 1997 to 2009. PLoS One. 2013;8(2):e56285. doi: 10.1371/journal.pone.0056285. Epub 2013 Feb 7. Erratum in: PLoS One. 2013;8(4). doi: 10.1371/annotation/e917b690-4e3a-4e90-8545-d4a172617b8f. PMID: 23409166; PMCID: PMC3567038.
**Measham, A. R., Rao, K. D., Jamison, D. T., Wang, J., & Singh, A.**	1999	Reducing infant mortality and fertility, 1975-1990 - Performance at all-India and state levels	Sample Registration Survey	Measham, Anthony R., et al. "Reducing infant mortality and fertility, 1975-1990: performance at all-India and state levels." *Economic and Political Weekly* (1999): 1359-1367.
**Million Death Study, C.**	2017	Changes in cause-specific neonatal and 1-59-month child mortality in India from 2000 to 2015: a nationally representative survey	Million Deaths Study	Million Death Study Collaborators. "Changes in cause-specific neonatal and 1-59-month child mortality in India from 2000 to 2015: a nationally representative survey." *Lancet (London, England)* vol. 390,10106 (2017): 1972-1980. doi:10.1016/S0140-6736(17)32162-1
**Munshi, V., Yamey, G., & Verguet, S.**	2016	Trends In State-Level Child Mortality, Maternal Mortality, And Fertility Rates In India	Sample Registration Survey	Munshi, Vidit et al. "Trends In State-Level Child Mortality, Maternal Mortality, And Fertility Rates In India." *Health affairs (Project Hope)* vol. 35,10 (2016): 1759-1763. doi:10.1377/hlthaff.2016.0552
**Narwal, R., & Gram, L.**	2013	Has the Rate of Reduction in Infant Mortality Increased in India Since the Launch of National Rural Health Mission? Analysis of Time Trends 2000	Sample Registration Survey	Narwal, Rajesh, and Lu Gram. "Has the Rate of Reduction in Infant Mortality Increased in India Since the Launch of National Rural Health Mission? Analysis of Time Trends 2000-2009 with Projection to 2015." *International journal of MCH and AIDS* vol. 2,1 (2013): 139-52. doi:10.21106/ijma.19
**Paul, V. K., Sankar, M. J., & Saini, S.**	2014	Trek to MDG 4: state of Indian States	Sample Registration Survey	Paul, Vinod K et al. "Trek to MDG 4: state of Indian States." *Indian journal of pediatrics* vol. 81,10 (2014): 993-9. doi:10.1007/s12098-013-1324-0

© 2024 Subramanian SV et al. *JAMA Network Open.*

**Prinja, S., Sharma, A., Nimesh, R., Sharma, V., Madan Gopal, K., Badgaiyan, N., Lakshmi, P., & Gupta, M.**	2021	Impact of National Health Mission on infant mortality in India: An interrupted time series analysis	Sample Registration Survey	Prinja, Shankar et al. "Impact of National Health Mission on infant mortality in India: An interrupted time series analysis." *The International journal of health planning and management* vol. 36,4 (2021): 1143-1152. doi:10.1002/hpm.3166
**Puranik, A., Binu, V. S., Biju, S., & Subba, S. H.**	2018	Spatio-temporal assessment of infant mortality rate in India	Sample Registration Survey	Puranik, Amitha et al. "Spatio-temporal assessment of infant mortality rate in India." *Indian journal of public health* vol. 62,1 (2018): 32-38. doi:10.4103/ijph.IJPH_356_16
**Pushkar.**	2012	Democracy and Infant Mortality in India's 'Mini-democracies': A Preliminary Theoretical Inquiry and Analysis	Sample Registration Survey, Census	Pushkar. "Democracy and Infant Mortality in India's 'Mini-democracies': A Preliminary Theoretical Inquiry and Analysis." *Journal of South Asian Development* 7.2 (2012): 109-137.
**Reddy, H., Pradhan, M. R., Ghosh, R., & Khan, A. G.**	2012	India's progress towards the Millennium Development Goals 4 and 5 on infant and maternal mortality	Sample Registration Survey	Reddy, Hanimi et al. "India's progress towards the Millennium Development Goals 4 and 5 on infant and maternal mortality." *WHO South-East Asia journal of public health* vol. 1,3 (2012): 279-289. doi:10.4103/2224-3151.207024
**Saikia, N., Shkolnikov, V. M., Jasilionis, D., & Chandrashekhar.**	2016	Trends and Sub-National Disparities in Neonatal Mortality in India from 1981 to 2011	Sample Registration Survey, Census	Saikia, Nandita, et al. "Trends and sub-national disparities in neonatal mortality in India from 1981 to 2011." *Asian Population Studies* 12.1 (2016): 88-107.

© 2024 Subramanian SV et al. *JAMA Network Open.*

eReferences

Bora JK, Saikia N. Neonatal and under-five mortality rate in Indian districts with reference to Sustainable Development Goal 3: An analysis of the National Family Health Survey of India (NFHS), 2015-2016. PLoS One. 2018 Jul 30;13(7):e0201125. doi: 10.1371/journal.pone.0201125. PMID: 30059555; PMCID: PMC6066210.

Kim R, Liou L, Xu Y, Kumar R, Leckie G, Kapoor M, Venkataramanan R, Kumar A, Joe W, Subramanian SV. Precision-weighted estimates of neonatal, Postneonatal and child mortality for 640 districts in India, National Family Health Survey 2016. J Glob Health. 2020 Dec;10(2):020405. doi: 10.7189/jogh.10.020405. PMID: 33110571; PMCID: PMC7568918.

Kumar C, Piyasa, Saikia N. An update on explaining the rural-urban gap in under-five mortality in India. BMC Public Health. 2022 Nov 16;22(1):2093. doi: 10.1186/s12889-022-14436-7. PMID: 36384563; PMCID: PMC9670513.

© 2024 Subramanian SV et al. *JAMA Network Open.*

eMethods 2: National Policies that may directly or indirectly influence childhood mortality in India.

Year of launch	Policy/Programme	Details
**1952**		It was launched to lower fertility rate and control population growth- maternal and child health was a small component of this plan
**1970**	National Nutritional Anaemia Prophylaxis Programme	It was launched to prevent nutritional anaemia in mothers and children- given one tablet of iron and folic acid containing 60 mg elementary iron which was raised to 100 mg elementary iron, however folic acid content remained the same (0.5 mg of folic acid) and children in the age group of 1-5 years are given one tablet of iron-containing 20 mg elementary iron (60 mg of ferrous sulphate and 0.1 mg of folic acid) daily for a period of 100 days.
**1970**	National Vitamin A Prophylaxis program	It was launched to prevent nutritional blindness. Under this strategy, every infant 6-11 months and children 1-5 years is to be administered vitamin A every 6 months. The recommended schedule is as follows: 6-11 months 1-5 years · one doze of 100,000 IU - 200,000 IU/6 months A child must receive a total of 9 oral doses of vitamin A by its firth birthday
**1970**	Special Nutrition Program	It provides supplementary feeding of about 300 calories and 10 grams of protein to preschool children and about 500 calories and 25 grams of protein to expect at and nursing mothers for six days a week.
**1974**	National Policy for Children	Included objective of general improvement in nutrition and diseases among mothers and children
**1975**	Anganwadi Services	Centres that provide services under the Integrated Child Development Service
**1975**	Targeted Public Distribution System	Provide food at lower costs to poor families through distribution centres- landmark policy of food security
**1977**	Family Welfare Programme	Integrated maternal and child health with family planning programs
**1978**	Expanded Programme on Immunization	It was launched to reduce morbidity and mortality from diphtheria, pertussis, tetanus, poliomyelitis and childhood tuberculosis by providing immunization services to all eligible children and pregnant women by 1990.
**1989**	Universal Immunization Programme	The program now consists of vaccination for 12 diseases- tuberculosis, diphtheria, pertussis (whooping cough), tetanus, poliomyelitis, measles, hepatitis B, diarrhea, Japanese encephalitis, rubella, pneumonia (Haemophilus influenzae type B) and Pneumococcal diseases (pneumococcal pneumonia and meningitis). Hepatitis B and Pneumococcal diseases were added to the UIP in 2007 and 2017 respectively.
**1992**	Child Survival and Safe Motherhood Programme	The policy had aimed to reduce maternal mortality to less than 2, infant mortality to less than 50 per 1000 live births; and child mortality (1 to 4 years of age) to below 10 by 2000
**1995**	Pulse Polio Programme	This has led to 100% immunization against polio, which was endemic to India
**1997**	Reproductive and Child Health services (for the unmet need of family planning)	It was launched to enable women to regulate fertility and to ensure safe pregnancy and childbirth- based on ICPD recommendations of 1994
**2003**	Integrated Management of Neonatal and Childhood Illnesses (IMNCI)	It aims to reduce preventable mortality, minimize illness and disability, and promote healthy growth and development of children under five years of age
**2005**	National Rural Health Mission	It aims to provide equitable, affordable, and quality health care to the rural population, especially vulnerable groups. It aimed to establish a fully functional, community-owned, decentralized health delivery system with inter-sectoral convergence at all levels, to ensure simultaneous action on a wide range of determinants of health such as water, sanitation, education, nutrition, social and gender equality
**2005**	Janani Suraksha Yojana	It integrates cash assistance with delivery and post-delivery care- and recognizes ASHA as a central link between the government and citizens for health
**2008**	Rashtriya Swashtya Bima Yojana	Rashtriya Swasthya Bima Yojana is a government-run health insurance program for the Indian poor. The scheme aims to provide health insurance coverage to the unrecognized sector workers belonging to the BPL category and their family members shall be beneficiaries under this scheme- maternity benefits and delivery care expenditure are included in the scheme
**2009**	Mother and Child Tracking System	Aims to leverage information technology for delivery of a full spectrum of healthcare and immunization services to pregnant women and children up to age of 5 years
**2009**	Navjaat Shishu Suraksha Karyakram (NSSK)	Aims to build the capacity of the doctors and nurses to provide essential newborn care around birth.
**2009**	Supplementary Nutrition Programme	It is one of the six services provided under the Integrated Child Development Services (ICDS) Scheme which is primarily designed to bridge the gap between the Recommended Dietary Allowance (FDA) and the Average Daily Intake (ADI).
**2011**	Janani Shishu Suraksha Karyakram	It entitles all pregnant women delivering in public health institutions to absolutely free and no expense delivery including Caesarean section

© 2024 Subramanian SV et al. *JAMA Network Open.*

**2011**	Home Based New Born Care	launched for accelerated reduction of Neonatal mortality and morbidity rates especially in rural, remote areas where access to care is largely unavailable or located faraway
**2013**	National Health Mission	Combines National Rural and Urban Health Missions- aims to provide equitable and quality primary healthcare services to the urban population with a special focus on slum and vulnerable sections of the society.
**2013**	Integrated Action Plan for Pneumonia and Diarrhea	It aims at ending preventable child deaths from pneumonia and diarrhea by 2025
**2013**	National Iron Plus Initiative	Under National Iron+ Initiative, the following age groups are covered for lifelong supplementation of Iron from the age of 6 months onwards: Bi-weekly 20 mg elemental iron and 100 micrograms (mcg) folic acid per ml of liquid formulation and age-appropriate de-worming for preschool children of 6-59 month
**2013**	Rashtriya Bal Swashta Krayakram	It aims to improve the overall quality of life of children enabling all children to achieve their full potential, and also provide comprehensive care to all the children in the community. This program involves screening children from birth to 18 years of age for 4 Ds- Defects at birth, Diseases, Deficiencies, and Development delays, spanning 32 common health conditions for early detection and free treatment and management, including surgeries at the tertiary level
**2013**	Safe Motherhood Booklets	Started along with National Health Mission to provide essential information to all pregnant women about maternal and child healthcare
**2013**	Weekly Iron Folic Acid Supplementation Programme	WIFS is an evidence-based programmatic response to the prevailing anemia situation amongst adolescent girls and boys through supervised weekly ingestion of IFA supplementation and biannual helminthic control. The long-term goal is to break the intergenerational cycle of anemia.
**2014**	India Newborn Action Plan	Six pillars of intervention packages impacting stillbirths and newborn health have been identified, which include: Preconception and antenatal care, Care during labour and childbirth, Immediate newborn care, Care of healthy newborns, Care of small and sick newborns, Care beyond newborn survival
**2014**	Lactation Management Centers	It is a center established in the health facility for the purpose of providing lactation support to all mothers within the health facility for collection, storage, and dispensing of a mother's own breast milk for consumption by her baby.
**2016**	Mothers Absolute Affection	Aims to revitalize efforts towards the promotion, protection and support of breastfeeding practices through health systems to achieve higher breastfeeding rates
**2016**	Pradhan Mantri Surakshit Matritva Abhiyaan	The program aims to provide assured, comprehensive and quality antenatal care, free of cost, universally to all pregnant women on the 9th of every month
**2017**	Labour room Quality improvement Initiative	The program will benefit every pregnant woman and newborn delivering in public health institutions. Program will improve quality of care for pregnant women in labour room, maternity Operation Theatre and Obstetrics Intensive Care Units (ICUs) & High Dependency Units (HDUs).
**2018**	Anemia Mukt Bharat	It has been designed to reduce the prevalence of anemia by 3 percentage points per year among children, adolescents and women in the reproductive age group (15–49 years), between the year 2018 and 2022
**2018**	POSHAN Abhiyaan	The programme, through use of technology, convergence and community involvement with a targeted approach strives to reduce the level of stunning, under-nutrition, Anemia and low birth weight in children, as also focus on adolescent girls, pregnant women and lactating mothers, thus holistically addressing malnutrition.
**2019**	Surakshit Matritva Aashwasan (SUMAN)	It aims to provide assured, dignified and respectful delivery of quality healthcare services at no cost and zero tolerance for denial of services to any woman and newborn visiting a public health facility in order to end all preventable maternal and newborn deaths and morbidities and provide a positive birthing experience
**2020**	Thalassemia Bal Sewa Yojna	It aims to provide a one-time cure opportunity for Haemoglobinopathies like Thalassaemia and Sickle Cell Disease for patients who have a matched family donor; and montly family icome is below INR 20000
**2022**	Intensified Diarrhoea Control Fortnight	It consists of a set of activities to be implemented in an intensified manner for prevention and control of deaths due to dehydration from diarrhoea across all States & UTs. These activities mainly include- the intensification of advocacy & awareness generation activities for diarrhea management, strengthening service provision for diarrhea case management, the establishment of ORS-Zinc corners, prepositioning of ORS by ASHA in households with under-five children, and awareness generation activities for hygiene and sanitation
**2022**	Intensified Mission Indradhanush	The scheme seeks to drive towards 90% full immunization coverage of India and sustain the same by the year 2022

© 2024 Subramanian SV et al. *JAMA Network Open.*

eMethods 3: Stata Codes to estimate Early-neonatal, Late-neonatal, Postneonatal and Child mortality rates across States/Union Territories of India, 1993 to 2021

```
* This program adds b19 to NFHS BR files before NFHS5

**************************************************************************

program define make_b19

* b1 and b2 are the month and year of birth
* v006 and v007 are the month and year of interview
* v016 is day of interview
* hw16 is day of birth, often missing
* set day or birth to 15 if missing,
*   but Include an exception if the birth was in the month of interview

* In the early surveys, year omits the first two digits and is 0 for 2000; must fix
replace v007=v007+1900 if v007>0 & v007<100
replace v007=2000      if v007==0

* Year of birth for the NFHS2 data omits the first two digits
replace b2=b2+1900 if b2>0 & b2<100
replace b2=2000    if b2==0

gen day_of_birth=hw16
replace day_of_birth=15 if hw16>31
replace day_of_birth=int((day_of_birth+hw16)/2) if v007==b2 & v006==b1 & day_of_birth<hw16

gen doi=mdy(v006,v016,v007)
gen dob=mdy(b1,day_of_birth,b2)

gen b19=int((doi-dob)/(365.25/12))

tab b19 if b19<=62

* Must be sure that there are no negative valuess of b19
replace b19=0 if b19<0
drop day* doi dob

end

**************************************************************************

* Execution begins here

* Specify workspace; revised data files will go here
cd ""

* Specify the path to the data files as a scalar
scalar spath=""

local lpath=spath

* Loop through the NFHS 1, 2, 3, 4. "pv" refers to the phase of the survey and version of the file

*foreach lpv in 23 42 52 74 {
foreach lpv in 23 42 52 74 {
use "`lpath'/IABR`lpv'FL.DTA", clear
make_b19
save IABR`lpv'FL_with_b19.DTA, replace
}
```

© 2024 Subramanian SV et al. *JAMA Network Open.*

```
/*********************************************************************************************************
Program:              CM_CHILD_WITH_NEONATAL28.do
Purpose:              Produce child mortality rates including 28-day neonatal and early neonatal
Data inputs:          BR dataset
Data outputs:         coded variables
Note:                 The program will produce a file "CM_rates_with_neonatal28_and_ci.dta"
*********************************************************************************************************/

clear
program drop _all
set more off

/*
*****************************************************************************************
PROGRAM TO PRODUCE UNDER 5 MORTALITY RATES FOR SPECIFIC WINDOWS OF TIME, WITH COVARIATES
*****************************************************************************************

The 5 standard rates (actually conditional probabilities) are as follows:

Neonatal mortality:     the probability of dying in the first month of life;
Postneonatal mortality: the difference between infant and neonatal mortality (similar to the
                            probability of dying during months 1 through 11, but different);
Infant mortality:       the probability of dying in the first year of life;
Child mortality:        probability of dying between the first and fifth birthday;
Under-five mortality:   the probability of dying before the fifth birthday.

Refer to these as q_nmr, q_pnmr, q_imr, q_cmr, and q_u5mr, respectively.

When these are multiplied by 1000, they are referred to as NMR, PNMR, IMR, CMR, and U5MR, respectively

The set of 5 is obtained from the set of 8 as follows:

q_nmr=q1
q_pnmr=q_imr-qnmr
q_imr=1-(1-q1)*(1-q2)*(1-q3)*(1-q4)
q_cmr=1-(1-q5)*(1-q6)*(1-q7)*(1-q8)
q_u5mr=1-(1-q1)*(1-q2)*(1-q3)*(1-q4)*(1-q5)*(1-q6)*(1-q7)*(1-q8) = 1-(1-q_imr)*(1-q_cmr)

This program produces a 28-day neonatal rate, plus early neonatal and late neonatal rates.
Those rates are just produced using a 60-month window of births, e.g. b19=1 to 61, with no censoring
  of neonatal deaths.
The difference between the 28-day q1 and the usual q1 is added into q2, for months 2-3. Because of the
  slippage in the definition of the window, the post neonatal rate does not necessarily go up. It may
  go either up or down, but by a small amount.

The new q's and those that differ from the usual construction are

q_nmr28 for days 0-27,
q_enmr for days 0-6,
qlnmr=qnmr28-q_enmr,
and q_pnmr28=q_imr-q_nmr28

Note that "28" is included in the labels q_nmr28 and q_pnmr28 because they will differ slightly from the
  usual construction.  "28" is NOT included in the labels for q_enmr and q_lnmr because they are completely
  new.  The same use of "28" is made for the notation of the rates per 1000.

GO TO THE REPEATED LINES OF ASTERISKS FOR THE BEGINNING OF THE EXECUTABLE STATEMENTS

*/

*****************************************************************************
* SUB-PROGRAMS OR ROUTINES BETWEEN HERE AND THE REPEATED LINES OF ASTERISKS
*****************************************************************************

program define setup

scalar stype=substr(sfn,3,2)

* For some surveys, such as IA23, the correct id here is v022, not v023
*gen stratumid=v023
gen stratumid=v022

* For some surveys, such as IA23, the correct id here is v001, not v021
*gen clusterid=v021
gen clusterid=v001
```

© 2024 Subramanian SV et al. *JAMA Network Open.*

```
* Specification of nageints, lengths of age intervals

/*
Standard specification but it can be changed:

i start_i  end_i  length_i
1    0        1      1
2    1        3      2
3    3        6      3
4    6       12      6
5   12       24     12
6   24       36     12
7   36       48     12
8   48       60     12

*/

scalar nageints=8

scalar length_1=1
scalar length_2=2
scalar length_3=3
scalar length_4=6
scalar length_5=12
scalar length_6=12
scalar length_7=12
scalar length_8=12

scalar start_1=0
local i=2
while `i'<=nageints+1 {
local iminus1=`i'-1
scalar start_`i'=start_`iminus1'+length_`iminus1'
local i=`i'+1
}

local i=1
while `i'<=nageints {
local iplus1=`i'+1
scalar end_`i'=start_`iplus1'
local i=`i'+1
}

rename v008 doi
quietly summarize doi [iweight=v005/1000000]
scalar doi_mean=r(mean)
gen wt=v005/1000000.
scalar v000_string=v000[1]
save temp1.dta, replace

end

******************************************************************************

program define start_month_end_month

* This routine calculates the end date and start date for the desired window of time
* It is called by prepare_child_file, which is called by make_risk_and_deaths
/*

For example,

scalar lw=-2
scalar uw=0

for a window from 0 to 2 years before the interview, inclusive
  (that is, three years)

lw is the lower end of the window and uw is the upper end.
(Remember that both are negative or 0.)

start_month is the cmc for the earliest month in the window and
end_month is the cmc for the latest month in the window

*/

* Section for "years before survey". lw and uw will be <=0
```

© 2024 Subramanian SV et al. *JAMA Network Open.*

```
if lw<=0 {

* coding that WILL NOT include the month of interview in the most recent interval; matches with DHS results

gen start_month=doi+12*lw-12
gen end_month=doi+12*uw-1
}

replace end_month=min(end_month,doi)

* calculate the reference date

quietly summarize start_month [iweight=v005/1000000]
scalar mean_start_month=r(mean)

summarize end_month [iweight=v005/1000000]
scalar mean_end_month=r(mean)

* Convert back to continuous time, which requires an adjustment of half a month (i.e. -1/24).
* This adjustment is not often made but should be.
scalar srefdate=1900-(1/24)+((mean_start_month+mean_end_month)/2)/12

end

******************************************************************************

program prepare_child_file

* This routine calculates dob, doi, dod_1, dod_2, and the start and end months
*  of each age interval for each child.

* This routine calls start_month_end_month and is called by make_risk_and_deaths

use temp1.dta, clear

start_month_end_month
gen KEEP=1

rename b3 dob

*drop if missing on birthdate
*************** NEW
*drop if dob==.
replace KEEP=0 if dob==.
***************

*drop if dob is later than the end of this time interval
*************** NEW
drop if dob>end_month
replace KEEP=0 if dob>end_month
***************

* b7 is given in single months up to 23 and then given as 24, 36, 48, 60, ...
*  but for the Uganda 2006 survey there are 2 births at 25, 1 at 26, 1 at 35

gen age_at_death=b7
replace age_at_death=. if age_at_death>end_month
replace age_at_death=. if age_at_death>59

*************** NEW
drop if dob+end_8<start_month-1
replace KEEP=0 if dob+end_8<start_month-1
***************

gen age_int_at_death=.
gen dod_1=.
gen dod_2=.

local i=1
while `i'<=nageints {
replace age_int_at_death=`i' if age_at_death>=start_`i' & age_at_death<=end_`i'-1
replace dod_1=dob+start_`i'  if age_int_at_death==`i'
replace dod_2=dob+end_`i'    if age_int_at_death==`i'
local i=`i'+1
}

drop if dod_2<start_month
replace dod_2=end_month if dod_1<= end_month & dod_2>end_month & end_month==doi-1
```
© 2024 Subramanian SV et al. *JAMA Network Open.*

```
save temp2.dta, replace

end

******************************************************************************
program define make_risk_and_deaths

prepare_child_file

use temp2.dta, replace

* Values of risk and died are calculated in the following loop.  It could be streamlined
*  somewhat but you must be careful or you will lose the match with DHS.

local i=1
while `i'<=nageints {
gen died`i'=.

* age interval is entirely in the time window
replace died`i'=1  if age_int_at_death==`i' &  dod_2<=end_month   & dod_1>=start_month

* age interval is partly in the time window and partly in the previous time window
replace died`i'=.5 if age_int_at_death==`i' &  dod_2>=start_month & dod_1< start_month

* age interval is partly in the time window and partly in the next time window
replace died`i'=.5 if age_int_at_death==`i' &  dod_2> end_month   & dod_1<=end_month

gen risk`i'=.

* age interval is entirely in the time window
replace risk`i'=1  if (age_int_at_death>=`i' | age_at_death==.) & dob+end_`i'<=end_month   &
dob+start_`i'>=start_month

* age interval is partly in the time window and partly in the previous time window
replace risk`i'=.5 if (age_int_at_death>`i'  | age_at_death==.) & dob+end_`i'>=start_month & dob+start_`i'<
start_month

* age interval is partly in the time window and partly in the next time window
replace risk`i'=.5 if (age_int_at_death>`i'  | age_at_death==.) & dob+end_`i'> end_month   &
dob+start_`i'<=end_month

* child dies in this interval
* age interval is partly in the time window and partly in the previous time window
replace risk`i'=.5 if age_int_at_death==`i' & end_`i'>=start_month & dob+start_`i'< start_month

* age interval is partly in the time window and partly in the next time window
replace risk`i'=.5 if age_int_at_death==`i' & end_`i'> end_month   & dob+start_`i'<=end_month

* Next line will change risk from 0 to .5 for a potential handful of cases in which died=.5 and risk=0;
*   it ensures a perfect match with the DHS programs.
replace risk`i'=.5 if died`i'==.5

* the preceding lines produce some values of died and risk that should be changed from missing to 0
replace died`i'=0 if died`i'==. & risk`i'>0 & risk`i'<=1
replace risk`i'=0 if risk`i'==. & died`i'>0 & died`i'<=1

replace risk`i'=1 if died`i'==1

* Everything is unweighted. Unweighted values are used in the logit model.
* The model includes weights in the specification.

local i=`i'+1
}

*************** NEW FOR THE 28-DAY NMR AND THE ENMR AND LNMR
* Define the five-year interval in terms of b19, e.g. 1 to 61 for 5 years if lw=-4
gen risk_B=1 if b19>=0 & b19<=1+(-lw+1)*12

* Define the neonatal, early neonatal, and late neonatal deaths with WHO definitions
gen     died_NMR28=0 if risk_B==1
replace died_NMR28=1 if risk_B==1 & b6<128

* Deaths in the first 7 days
gen     died_ENMR=0 if risk_B==1
replace died_ENMR=1 if risk_B==1 & b6<107

* Deaths in the first 28 days but not in the first 7 days
```
© 2024 Subramanian SV et al. *JAMA Network Open.*

```
gen     died_LNMR=0 if risk_B==1
replace died_LNMR=1 if risk_B==1 & died_NMR==1 & died_ENMR==0
***************

sort caseid bidx

save risk_and_deaths.dta, replace

* This is a child-level file that includes a line for every child, with values of died and risk for each
*   of the eight basic age intervals.

end

******************************************************************************

program define calculate_q_no_ci

* simple calculation of rates with division: q=died/risk

foreach lc of global gcovars_temp {

use risk_and_deaths.dta, clear

keep died* risk* v005 `lc'

collapse (sum) died* risk* [iweight=v005/1000000], by(`lc')
local llabel : variable label `lc'
scalar slabel_`lc'="`llabel'"
levelsof `lc', local(levels_`lc')

forvalues lr=1/8 {
gen q`lr'=died`lr'/risk`lr'
}

********* NEW
gen q_enmr=died_ENMR/risk_B
gen q_lnmr=died_LNMR/risk_B
gen q_nmr28=died_NMR/risk_B
*********

drop died* risk*

gen v_run=srun_no_ci
gen str10 variable="`lc'"
gen value=`lc'
gen str30 variable_label=slabel_`lc'
gen str30 value_label="."
order v_run variable value variable_label value_label q*

foreach li of local levels_`lc' {
local lname : label (`lc') `li'
replace value_label="`lname'" if `lc'==`li'
}

gen v_lw=lw
gen v_uw=uw
gen v_mean_doi=smean_doi
gen v_refdate=srefdate

gen NMR=q1
gen IMR  =1-(1-q1)*(1-q2)*(1-q3)*(1-q4)
gen PNMR=IMR-NMR
gen CMR  =1-(1-q5)*(1-q6)*(1-q7)*(1-q8)
gen U5MR =1-(1-IMR)*(1-CMR)

foreach lt in N PN I C U5 {
replace `lt'MR=1000*`lt'MR
}

************* NEW
gen ENMR=q_enmr
gen LNMR=q_lnmr
gen NMR28=q_nmr

foreach lt in ENMR LNMR NMR28 {
replace `lt'=1000*`lt'
}
gen PNMR28=IMR-NMR28
```
© 2024 Subramanian SV et al. *JAMA Network Open.*

```
*************

drop `lc'

if srun_no_ci>1 {
append using partial_results_no_ci.dta
}

save partial_results_no_ci.dta, replace
scalar srun_no_ci=srun_no_ci+1
}

*order v_* variable value ENMR LNMR NMR28 PNMR28 NMR PNMR IMR CMR U5MR

replace variable_label=strproper(variable_label)
replace value_label=strproper(value_label)
list variable value variable_label value_label ENMR LNMR NMR28 PNMR28 NMR PNMR IMR CMR U5MR, table clean
rename v_* *
sort run
drop run
save partial_results_no_ci.dta, replace

end

******************************************************************************

program define calculate_q_with_ci

* The UNWEIGHTED deaths and risk are used, with adjustments for weights, clusters, and strata within the model.

use risk_and_deaths.dta, clear

* For the India surveys, v024 is needed for a unique id
*egen motherid=group(v001 v002 v003)
egen motherid=group(v001 v002 v003 v024)
egen childid=group(motherid bidx)

keep *id doi b6 b7 died* risk* v005 KEEP v024 $gcovars_temp

***********************
quietly reshape long died risk, i(childid) j(age)
***********************

* Construct dummy variables for ALL age groups ("noomit" is crucial!).
xi, noomit i.age
rename _I* *

* died is coded "." if there is no risk in the age/time interval; can drop such lines
*drop if died==.

***********************
svyset clusterid [pweight=v005], strata(stratumid) singleunit(centered)

* If the stratum adjustment is not working, use a version that omits it
svyset clusterid [pweight=v005]

svyset [pweight=v005], strata(stratumid) singleunit(centered)


***********************

save temp0.dta, replace

foreach lc of global gcovars_temp {
local llabel : variable label `lc'
scalar slabel_`lc'="`llabel'"
scalar sc="`lc'"

levelsof `lc', local(levels_`lc')
  foreach li of local levels_`lc' {

local lname : label (`lc') `li'
scalar sname_`lc'_`li'="`lname'"

scalar si=`li'
scalar list sc si
gen subpop_code=1 if `lc'==`li' & KEEP==1
```

© 2024 Subramanian SV et al. *JAMA Network Open.*

```
************* NEW
* Do not alter the usual glm model. We want to include the IMR, CMR, and U5MR as usually calculated
* The early neonatal, late neonatal, and neonatal rates will come from a separate model.
* The Postneonatal rate will be adjusted so that it is the difference between IMR and NMR28
* Save as scalars.

foreach lt in ENMR LNMR NMR28 {

***********************
svy, subpop(subpop_code): glm died_`lt', family(binomial risk_B) link(logit) iter(50)
***********************

matrix T=r(table)
scalar P=T[1,1]
scalar L=T[5,1]
scalar U=T[6,1]
scalar s`lt'  =exp(P)/(1+exp(P))
scalar s`lt'_L=exp(L)/(1+exp(L))
scalar s`lt'_U=exp(U)/(1+exp(U))
}
*************

* Estimate the 8 q's
***********************
svy, subpop(subpop_code): glm died age_*, nocons family(binomial risk) link(logit) iter(50)
***********************

*tab died risk if age_1==1

* Get the 5 rates, with c.i., from the 8 q's, using nlcom
forvalues la=1/8 {
local lb`la'="_b[died:age_`la']"

* terms for 1-q = 1/(1+exp(b))
local lt="_b[died:age_`la']"
local lterm`la'="1/(1+exp(`lt'))"
}

* The standard 5 rates are nmr, pnmr, imr, cmr, u5mr, which will be referred to as 1, 2, 3, 4, 5

local lr1 ="1-`lterm1'"
local lr2 ="`lterm1'*(1-`lterm2'*`lterm3'*`lterm4')"
local lr3 ="1-(`lterm1'*`lterm2'*`lterm3'*`lterm4')"
local lr4 ="1-(`lterm5'*`lterm6'*`lterm7'*`lterm8')"
local lr5 ="1-(`lterm1'*`lterm2'*`lterm3'*`lterm4'*`lterm5'*`lterm6'*`lterm7'*`lterm8')"

quietly nlcom (`lr1') (`lr2') (`lr3') (`lr4') (`lr5'), post iter(50)
* There does not seem to be a saved table r(table) after nlcom, so we must calculate ci's from B and V
* This approach produces symmetric confidence intervals, unfortunately
matrix B=e(b)
matrix V=e(V)

* Save a line of results
clear
set obs 1
gen v_run=srun_with_ci
gen str10 variable=sc
local lc=sc
gen str30 variable_label=slabel_`lc'

gen value=si
local li=si
gen str30 value_label=sname_`lc'_`li'

gen v_lw=lw
gen v_uw=uw
gen v_mean_doi=smean_doi
gen v_refdate=srefdate

************* NEW
foreach lt in ENMR LNMR NMR28 {
gen `lt'  =1000*s`lt'
gen `lt'_L=1000*s`lt'_L
gen `lt'_U=1000*s`lt'_U
}
*************

forvalues lr=1/5 {
```
© 2024 Subramanian SV et al. *JAMA Network Open.*

```
gen v_r`lr'_b=B[1,`lr']
gen v_r`lr'_V=V[`lr',`lr']
}

if srun_with_ci>1 {
append using partial_results_with_ci.dta
}

save partial_results_with_ci.dta, replace
scalar srun_with_ci=srun_with_ci+1
use temp0.dta, clear
  }
}

use partial_results_with_ci.dta, clear
rename v_* *
sort run
drop run
* Compressed code for constructing the rates, with c.i., from the b's and V's
local ln=1
foreach lt in NMR PNMR IMR CMR U5MR {
gen `lt'  =1000*r`ln'_b
gen `lt'_L=1000*(r`ln'_b - 1.96*sqrt(r`ln'_V))
gen `lt'_U=1000*(r`ln'_b + 1.96*sqrt(r`ln'_V))
local ln=`ln'+1
}

* Calculate the new PNMR, called PNMR28, as IMR-NMR28. Give it the same se as PNMR.
gen PNMR28=IMR-NMR28
gen PNMR28_L=PNMR28-1.96*sqrt(r2_V)
gen PNMR28_U=PNMR28+1.96*sqrt(r2_V)

drop r*_b r*_V
save partial_results_with_ci.dta, replace

end

**********************************************************

program define final_file_save

* The variables in the results will be equenced as in each version of gcovars_temp

if sinclude_ci==0 {
use partial_results_no_ci.dta, clear
}

if sinclude_ci==1 {
use partial_results_with_ci.dta, clear
}

format *MR* %6.2f
format refdate mean_doi %7.2f
order lw uw refdate mean_doi variable value variable_label value_label ENMR* LNMR* NMR28* PNMR28* IMR* CMR*
U5MR*


sort lw uw variable value
list lw uw refdate mean_doi variable value variable_label value_label ENMR LNMR NMR28 PNMR28 NMR PNMR IMR CMR
U5MR, table clean

if sinclude_ci==1 {
list lw uw refdate mean_doi variable value variable_label value_label ENMR_L LNMR_L NMR28_L PNMR28_L NMR_L
PNMR_L IMR_L CMR_L U5MR_L, table clean
list lw uw refdate mean_doi variable value variable_label value_label ENMR_U LNMR_U NMR28_U PNMR28_U NMR_U
PNMR_U IMR_U CMR_U U5MR_U, table clean
}

* Also list the estimates in a way that allows easy comparison with tables 8.1, 8.2, and 8.3

* Table 8.1 and the totals rows in tables 8.2 and 8.3
gen neglw=-lw
sort neglw
drop neglw
list lw uw refdate mean_doi variable value variable_label value_label ENMR LNMR NMR28 PNMR28 IMR CMR U5MR if
variable=="total", table clean
export excel Chapter8.xlsx, sheet("Table 8.1") sheetreplace firstrow(var)
```

© 2024 Subramanian SV et al. *JAMA Network Open.*

```
if sinclude_ci==1 {
* List L and U for table 8.1 and the totals rows for tables 8.2 and 8.3
* These 95% confidence intervals are slightly different from those given in Appendix B of the final report
* They are calculated to be symmetric on the scale of logit(q). Those in Appendix B are symmetric on the scale
of q.
list lw uw refdate mean_doi variable value variable_label value_label NMR28_L PNMR28_L IMR_L CMR_L U5MR_L if
variable=="total", table clean
export excel Chapter8.xlsx, sheet("Table 8.1L") sheetreplace firstrow(var)
list lw uw refdate mean_doi variable value variable_label value_label NMR28_U PNMR28_U IMR_U CMR_U U5MR_U if
variable=="total", table clean
export excel Chapter8.xlsx, sheet("Table 8.1U") sheetreplace firstrow(var)
}

* Table 8.2
sort variable value
list lw uw refdate mean_doi variable value variable_label value_label NMR28 PNMR28 IMR CMR U5MR if
variable=="child_sex" | variable=="v025", table clean
export excel Chapter8.xlsx, sheet("Table 8.2") sheetreplace firstrow(var)

* Table 8.3
* Any survey-specific panels in table 8.3 would require adjustments to this program
* In the reports Table 8.3 does not include a totals row but one is given here
list lw uw refdate mean_doi variable value variable_label value_label NMR28 PNMR28 IMR CMR U5MR if lw==-9 &
uw==0, table clean
export excel Chapter8.xlsx, sheet("Table 8.3") sheetreplace firstrow(var)

* drop q*

if sinclude_ci==0 {
save CM_rates_with_neonatal28.dta, replace
}

if sinclude_ci==1 {
save CM_rates_with_neonatal28_and_ci.dta, replace
}


* optional--erase the working files
*erase partial_results.dta
erase risk_and_deaths.dta
erase temp1.dta
erase temp2.dta
erase BRtemp.dta
end

******************************************************************************

program define recodes

* Routine to recode or construct covariates

* Be sure that the covariates are included in the the original save and reshape commands

* Example: combine codes 2 and 3 of v106, Education

*gen v106r=v106
*replace v106r=2 if v106==3

gen total=1
label variable total "Total"
label define total 1 "Total"
label values total total

gen child_sex=b4
label variable child_sex "Sex of child"
label define child_sex 1 "Male" 2 "Female"
label values child_sex child_sex

* mother's age at birth (years): <20, 20-29, 30-39, 40-49
gen months_age=b3-v011
gen mo_age_at_birth=1 if months_age<20*12
replace mo_age_at_birth=2 if months_age>=20*12 & months_age<30*12
replace mo_age_at_birth=3 if months_age>=30*12 & months_age<40*12
replace mo_age_at_birth=4 if months_age>=40*12 & months_age<50*12
drop months_age
label variable mo_age_at_birth "Mother's age at birth"
label define mo_age 1 "<20" 2 "20-29" 3 "30-39" 4 "40-49"
label values mo_age_at_birth mo_age
```
© 2024 Subramanian SV et al. *JAMA Network Open.*

```
* birth order: 1, 2-3, 4-6, 7+
gen birth_order=1
replace birth_order=2 if bord>1
replace birth_order=3 if bord>3
replace birth_order=4 if bord>6
replace birth_order=. if bord==.
label variable birth_order "Birth order"
label define birth_order 1 "1" 2 "2-3" 3 "4-6" 4 "7+"
label values birth_order birth_order

* preceding birth interval (years): <2, 2, 3, 4+
gen prev_bint=1
replace prev_bint=2 if b11>23
replace prev_bint=3 if b11>35
replace prev_bint=4 if b11>47
replace prev_bint=. if b11==.
label variable prev_bint "Preceding birth interval (years)"
label define prev_bint 1 "<2 years" 2 "2 years" 3 "3 years" 4 "4+ years"
label values prev_bint prev_bint

* birth size: small or very small, average or larger
gen birth_size=1
replace birth_size=2 if m18<=3
replace birth_size=. if m18>5
label variable birth_size "Birth size"
label define birth_size 1 "Small or very small" 2 "Average or larger"
label values birth_size birth_size

save, replace

end

*******************************************************************************

program define main

use BRtemp.dta, clear
quietly summarize v008 [iweight=v005/1000000]
scalar smean_doi=1900-(1/24)+(r(mean))/12

* You can run two versions of calculate_q
* calculate_q_no_ci is much faster
* calculate_q_with_ci is used to produce tables 8.1 to 8.4

setup
*quietly setup
quietly recodes
quietly make_risk_and_deaths

if sinclude_ci==0 {
calculate_q_no_ci
use partial_results_no_ci.dta, clear
}

if sinclude_ci==1 {
calculate_q_with_ci
use partial_results_with_ci.dta, clear
}

end

* EXECUTION BEGINS HERE

* Input file should be BR

* srun is a counter used for the construction of the output file
scalar srun_no_ci=1
scalar srun_with_ci=1

* IDENTIFY WHERE YOU WANT THE LOG AND OUTPUT FILES TO GO AND THE NAME OF THE LOG FILE

* Specify the path to the log file and the output files as a scalar
* This path was defined in the main file. If you do not use the main file you need to provide it here.

* scalar soutpath="C:/Users//$user/ICF/Analysis - Shared Resources/Code/DHS-Indicators-Stata/Chap08_CM"
* local loutpath=soutpath
* cd "`loutpath'"
```
© 2024 Subramanian SV et al. *JAMA Network Open.*

```
* Specify the name of the log file as a scalar
* set logtype text
* scalar slogfile="DHS_U5_rates_log.txt"
* local llogfile=slogfile
* log using "`llogfile'",replace

***********************
* Specify the path to the input data as a scalar
* datapath is defined in the main file. If you do not use the main file you need to provide it here.
scalar spath=" "
local lpath=spath
***********************

***********************
* Specify the file name as a scalar.
* The survey is defined in the main file. If you do not use the main file you need to provide it here.
scalar sfn=" "
***********************

**************************************************************
* SPECIFY WHETHER YOU DO OR DO NOT WANT CONFIDENCE INTERVALS
* THE VERSION WITH NO CI IS MUCH FASTER AND IS THE DEFAULT
* REMOVE THE ASTERISK IN FRONT OF THE SECOND LINE TO INCLUDE CI
* PUT AN ASTERISK IN FRONT OF THE SECOND LINE TO OMIT CI
* scalar sinclude_ci=0
scalar sinclude_ci=1
**************************************************************

local lfn=sfn
use "`lpath'//`lfn'", clear

* IMPORTANT: Reduce to the variables that are needed. b6 and b19 are needed for this version.
* gcovars includes total, so construct a temporary version and then delete it because it is constructed later
gen total=1
keep caseid v000 v001 v002 v003 v005 v008 v011 v201 bidx* bord* b3* b4* b6* b7* b11* b19* m18* v021 v022 v023
v024 $gcovars
rename caseid original_caseid
gen caseid=_n
save BRtemp.dta, replace


* The following setup is for the standard tables but can be altered
* lw and uw describe the window of time as the lower and upper ends of "years ago"

use BRtemp.dta, clear
scalar lw=-4
scalar uw=0
global gcovars_temp "total v024"
main

/*
use BRtemp.dta, clear
scalar lw=-9
scalar uw=-5
global gcovars_temp "total"
main

use BRtemp.dta, clear
scalar lw=-14
scalar uw=-10
global gcovars_temp "total"
main

use BRtemp.dta, clear
scalar lw=-9
scalar uw=0
global gcovars_temp "mo_age_at_birth birth_order prev_bint birth_size $gcovars"
main
*/

* THE NEXT LINE IS ESSENTIAL AT THE END OF THE RUN

final_file_save


******************************************************************************
```

© 2024 Subramanian SV et al. *JAMA Network Open.*
