# Supplementary material for: Early-Neonatal, Late-Neonatal, Postneonatal, and Child Mortality Rates Across India, 1993-2021
Source: JAMA Netw Open. 2024 May 10;7(5):e2410046. doi: 10.1001/jamanetworkopen.2024.10046 (PMC11087840; doi:10.1001/jamanetworkopen.2024.10046)
Supplement: Supplement 2. — Data Sharing Statement [file jamanetwopen-e2410046-s002.pdf]

Data Sharing Statement

Subramanian. Early-Neonatal, Late-Neonatal, Postneonatal, and Child Mortality Rates Across India, 1993-2021. *JAMA Netw Open*. Published May 10, 2024.
doi:10.1001/jamanetworkopen.2024.10046

Data
Data available: No

Additional Information
Explanation for why data not available: The study is based on publicly available data and can be accessed from https://dhsprogram.com/data/available-datasets.cfm. The underlying data tables used in this analysis can be accessed from the interactive dashboard linked here: https://geographicinsights.iq.harvard.edu/State-Child-Mortality.
